# Supplementary material for: The chirality of the mitotic spindle provides a mechanical response to forces and depends on microtubule motors and augmin
Source: Curr Biol. 2022 Jun 6;32(11):2480–2493.e6. doi: 10.1016/j.cub.2022.04.035 (PMC9235856; doi:10.1016/j.cub.2022.04.035)
Supplement: Document S1. Figures S1–S6 [file mmc1.pdf]

Current Biology, Volume *32*

Supplemental Information

The chirality of the mitotic spindle provides a mechanical response to forces and depends on microtubule motors and augmin

Monika Trupinić, Barbara Kokanović, Ivana Ponjavić, Ivan Barišić, Siniša Šegvić, Arian Ivec, and Iva M. Tolić

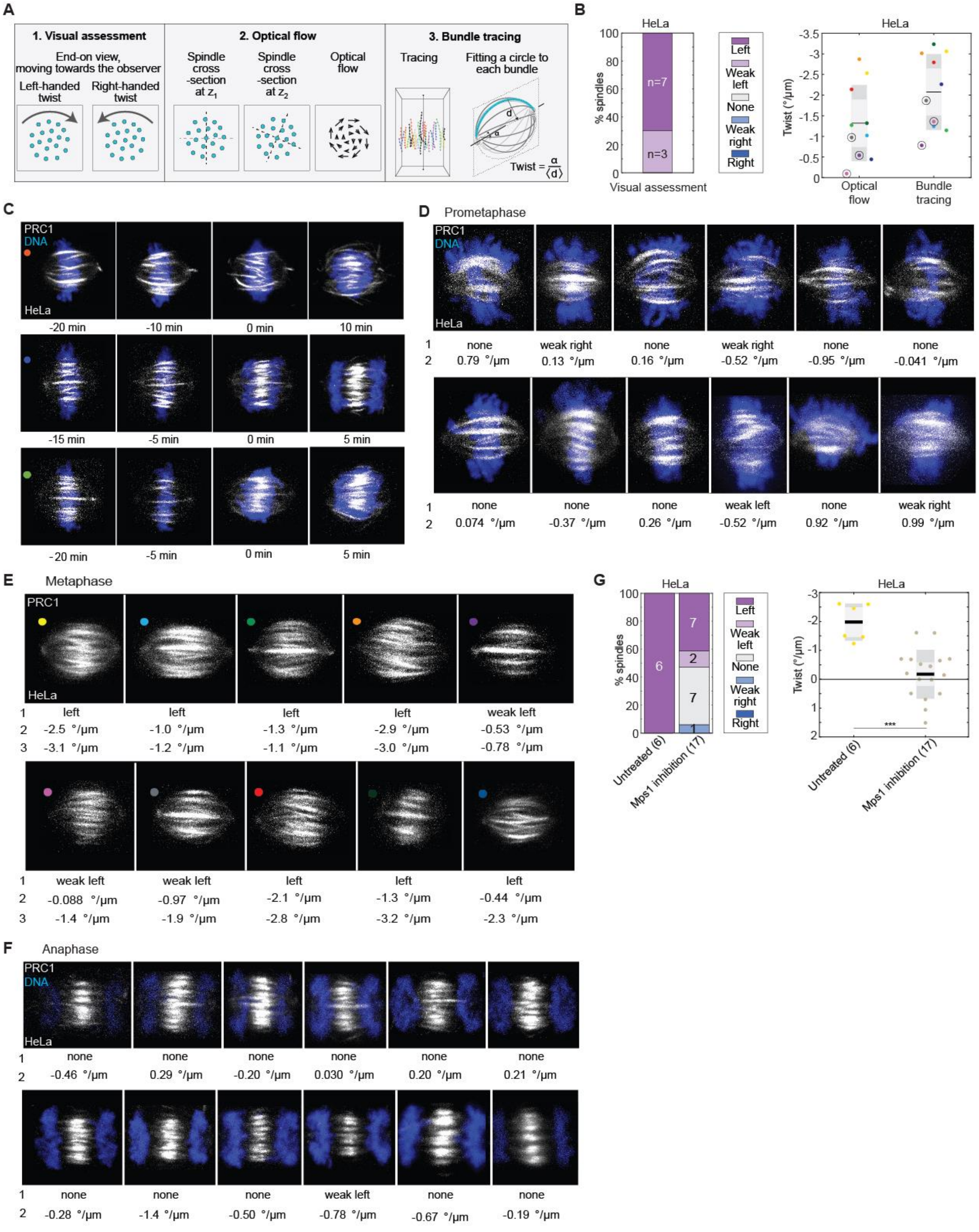

1 = visual assessment   2 = optical flow   3 = bundle tracing

Figure S1. Methods for calculating twist and progression of twist during mitosis in HeLa cells. Related to Figure 1.

(A) Schemes of three methods used to measure spindle twist: visual assessment (1), optical flow (2) and bundle tracing (3).

(B) Comparison of the twist for 10 spindles calculated with three different methods. On the left, visual assessment graph represents percentages of spindles showing left, right, weak left, weak right or no twist as described in the legend. On the right, graph shows twist calculated with optical flow and bundle tracing methods; each color represents one cell; circled and un-circled data correspond to the 'weak left' and 'left' data from the visual assessment graph, respectively. Note that the weak left twist in the visual assessment graph corresponds to the range of approximately -1 to -2 °/μm in the bundle tracing method followed along the 5 μm of the bundle length, which corresponds to the rotation of 5-10° in the clockwise direction in the end-on view of the spindle. The black line shows the mean; the light and dark grey areas mark 95% confidence interval on the mean and standard deviation, respectively. Same cells were used to calculate the data for both methods. Experiments were performed on the HeLa-Kyoto BAC cells stably expressing PRC1-GFP (n=10; raw data taken and re-calculated from [S1]).

(C) Microscope images of HeLa cells' spindles in time. Each colored dot represents one spindle's progression through mitosis in time, and each color matches the color of the spindle's data in the graph in Figure 1C. Three individual examples are shown. Microtubule bundles are shown in grey (PRC1-GFP) and DNA in blue (SiR-DNA dye). Images are shown in maximum z-projections. Experiments were performed on the HeLa-Kyoto BAC cells stably expressing PRC1-GFP. Related to Figure 1C.

(D-F) Microscope images of individual **s**pindles of HeLa cells in different phases of mitosis. Examples of spindles in prometaphase (D), metaphase (E) and anaphase (F) are shown with their twist values. Twist was determined with the visual assessment method and the optical flow, marked 1 and 2, respectively. For metaphase spindles twist was also calculated using bundle tracing method, marked 3. Data from these cells was used in the graphs in Figure 1D. Data from metaphase cells was also used in Figure. S1B. Microtubule bundles are shown in grey (PRC1-GFP) and DNA in blue (SiR-DNA dye). Images are shown in maximum z-projections. Experiments were performed on the HeLa-Kyoto BAC cells stably expressing PRC1-GFP. Related to Figure 1B.

(G) Graphs showing twist values after the inhibition of Mps1 in HeLa cells. On the left, visual assessment graph represents percentages of spindles showing left, right, weak left, weak right or no twist as described in the legend. On the right, graph shows twist calculated with the optical flow method. The black line shows the mean; the light and dark grey areas mark 95% confidence interval on the mean and standard deviation, respectively. ***$p<0.001$ (Student's t-test). Experiments were performed on the HeLa-Kyoto BAC cells stably expressing PRC1-GFP.

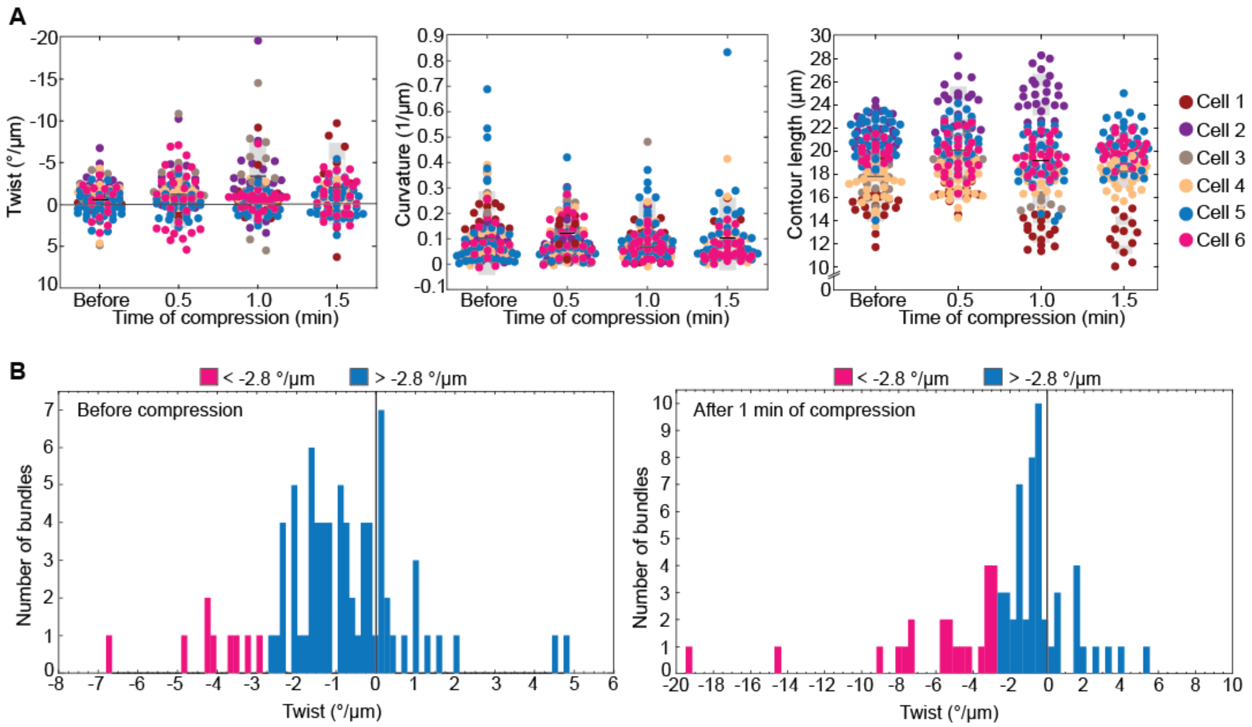

Figure S2. Twist, curvature and contour length of microtubule bundles in spindles compressed by an external force. Related to Figure 2.

(A) On the left, graph show the change of the twist before and up to 1.5 min after the compression. In the middle, graph shows the change of the curvature before and up to 1.5 min after the compression. On the right, graph shows the change of length of the bundle contours before and up to 1.5 min after the compression. Each color represents one cell, as described in the legend; dots represent bundles; the black line shows the mean; the light and dark grey areas mark 95% confidence interval on the mean and standard deviation, respectively. Experiments were performed on the HeLa-Kyoto BAC cells stably expressing PRC1-GFP. Related to Figures 2C-G.

(B) Histograms of twist values before (left) and 1 minute after the compression (right). Colors magenta and blue represent bundles with twist values lower than -2.8 °/μm and above -2.8 °/μm (one standard deviation away from the mean twist before compression), respectively. Note that the distribution shifted towards more negative values upon compression. The twist was smaller than -2.8 °/μm (corresponding to strong left-handed twist) for 9 out of 80 bundles (11.3% ± 3.5%) before compression, whereas after compression this was the case for 21 out of 73 bundles (28.8% ± 5.3%).

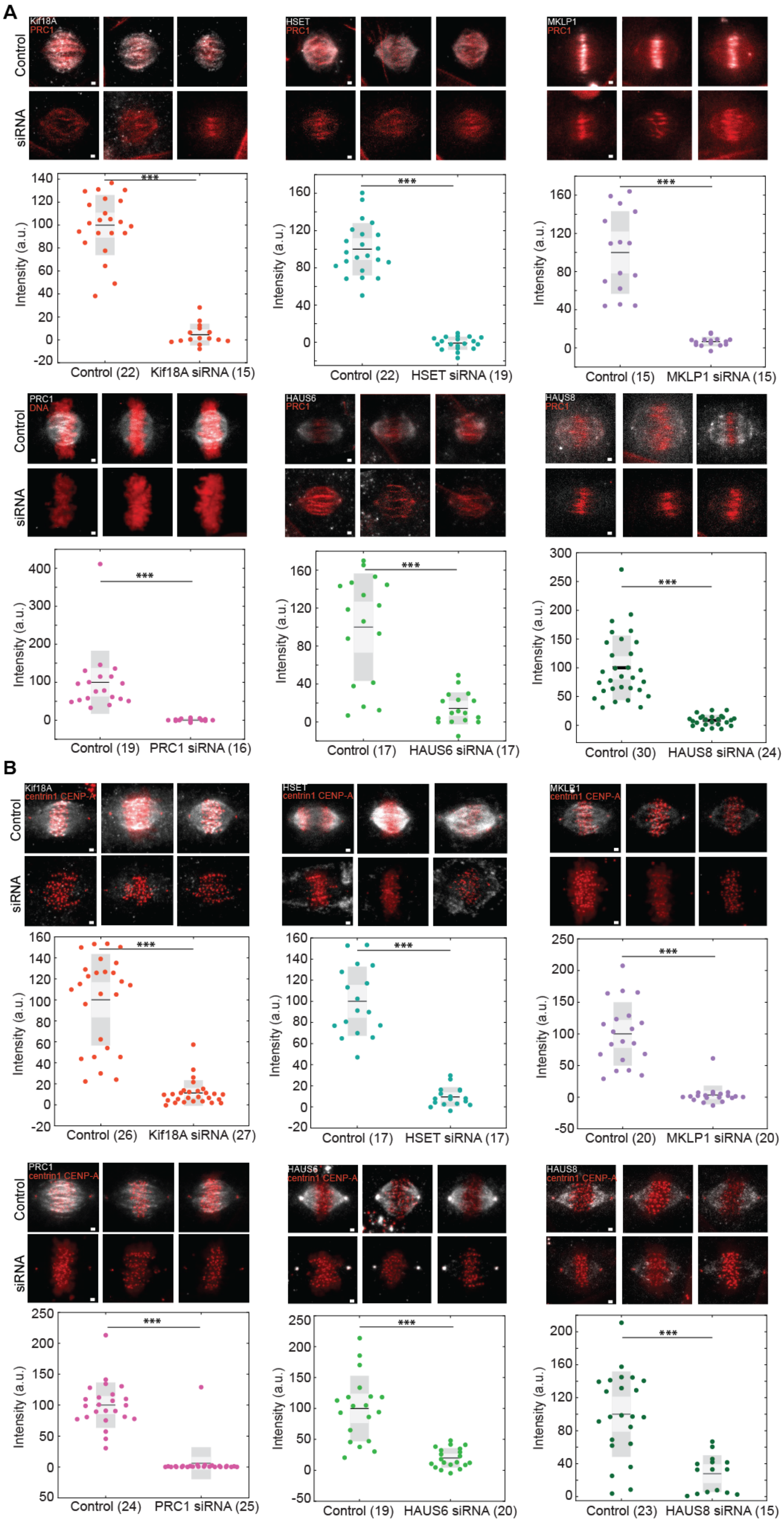

A
Kif18A
PRC1
Control
siRNA
HSET
PRC1
MKLP1
PRC1
Intensity (a.u.)
***
Control (22)
Kif18A siRNA (15)
Control (22)
HSET siRNA (19)
Control (15)
MKLP1 siRNA (15)
PRC1
DNA
HAUS6
PRC1
HAUS8
PRC1
Control (19)
PRC1 siRNA (16)
Control (17)
HAUS6 siRNA (17)
Control (30)
HAUS8 siRNA (24)
B
Kif18A
centrin1 CENP-A
HSET
centrin1 CENP-A
MKLP1
centrin1 CENP-A
Control (26)
Kif18A siRNA (27)
Control (17)
HSET siRNA (17)
Control (20)
MKLP1 siRNA (20)
PRC1
centrin1 CENP-A
HAUS6
centrin1 CENP-A
HAUS8
centrin1 CENP-A
Control (24)
PRC1 siRNA (25)
Control (19)
HAUS6 siRNA (20)
Control (23)
HAUS8 siRNA (15)

Figure S3. Immunofluorescence images of spindles in RPE1 and HeLa cells after protein perturbations. Related to Figures 3 and 4.

(A) Immunofluorescence of HeLa cells' spindles after perturbation of spindle-associated proteins. Examples of three spindles for every perturbation of spindle-associated proteins and their controls: Kif18A depletion, MKLP1 depletion, HSET depletion, PRC1 depletion, HAUS6 and HAUS8 depletion, in that order. Microtubule bundles are shown in grey (proteins of interest) and DNA (dyed with DAPI in non-transfected HeLa) or PRC1 (in HeLa-Kyoto BAC cells stably expressing PRC1-GFP) in red. Images are shown in maximum z-projections. Graphs show intensities of protein of interest in control cells and cells treated with siRNA. ***$p<0.001$ (Student's t-test). Experiments were performed on the non-transfected HeLa cell line (for the depletion of PRC1 and its control) and HeLa-Kyoto BAC cells stably expressing PRC1-GFP (for the rest of the treatments). Related to Figures 3B and 4B.

(B) Immunofluorescence of RPE1 cells' spindles after perturbation of spindle-associated proteins. Examples of three spindles for every perturbation of spindle-associated proteins and their controls: Kif18A depletion, HSET depletion, MKLP1 depletion, PRC1 depletion, HAUS6 and HAUS8 depletion, in that order. Microtubule bundles are shown in grey (proteins of interest) and kinetochores/centrosomes in red. Images are shown in maximum z-projections. Graphs show intensities of protein of interest in control cells and cells treated with siRNA. ***$p<0.001$ (Student's t-test). Experiments were performed on the hTERT-RPE1 cells, permanently transfected and stabilized using CENP-A-GFP and centrin1-GFP. Related to Figures 3C and 4C.

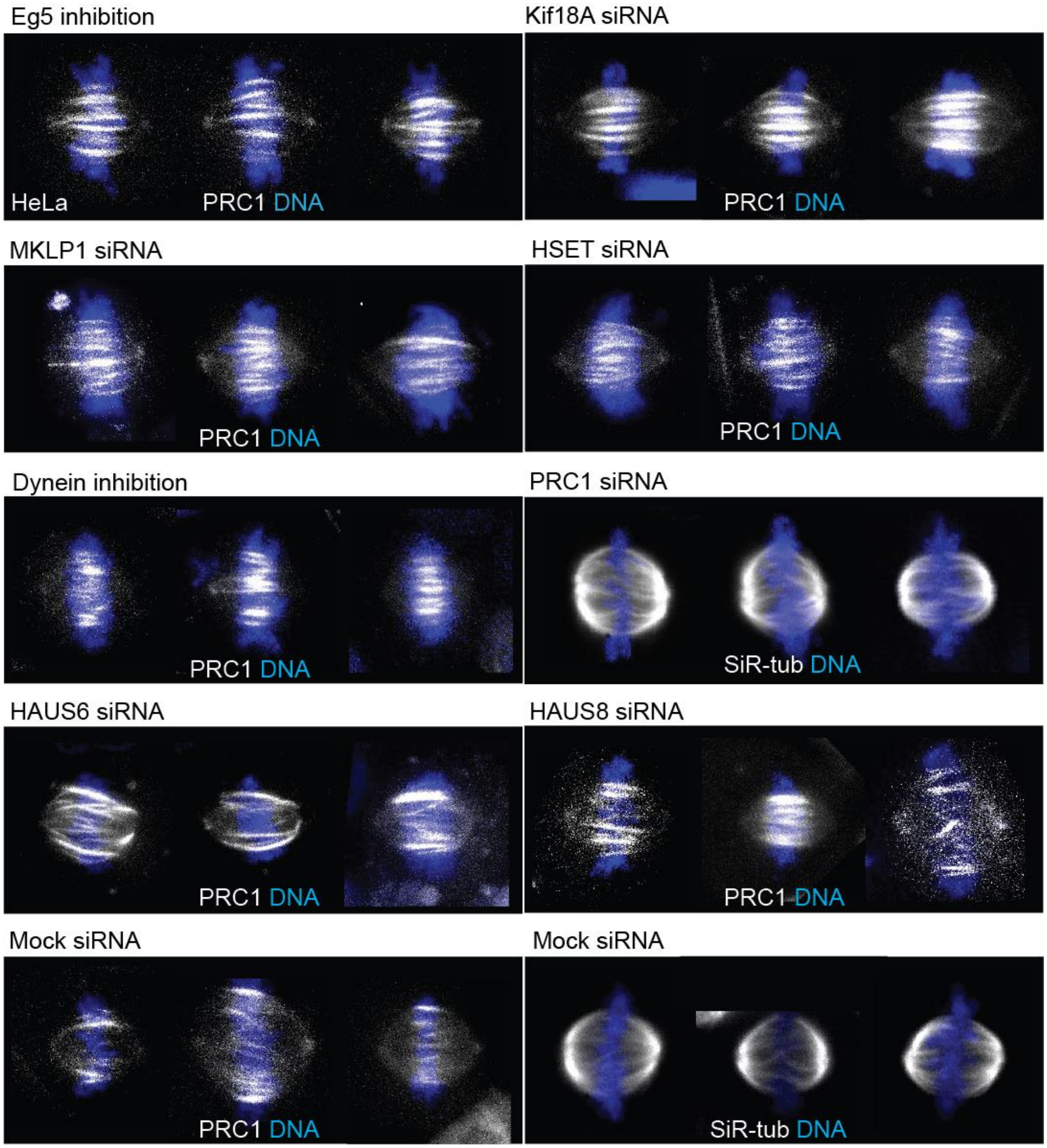
Eg5 inhibition
HeLa
PRC1 DNA
Kif18A siRNA
PRC1 DNA
MKLP1 siRNA
PRC1 DNA
HSET siRNA
PRC1 DNA
Dynein inhibition
PRC1 DNA
PRC1 siRNA
SiR-tub DNA
HAUS6 siRNA
PRC1 DNA
HAUS8 siRNA
PRC1 DNA
Mock siRNA
PRC1 DNA
Mock siRNA
SiR-tub DNA

Figure S4. Spindles of HeLa cells after perturbation of spindle-associated proteins. Related to Figures 3 and 4.

Examples of three spindles for every perturbation of spindle-associated proteins: Eg5 inhibition, Kif18A depletion, MKLP1 depletion, HSET depletion, dynein inhibition, PRC1 depletion, HAUS6 and HAUS8 depletion and mock controls. Data was used in the graphs in Figures. 3B and 4B. Microtubule bundles are shown in grey (SiR-tubulin in non-transfected HeLa and PRC1-GFP in HeLa-Kyoto BAC cell line) and DNA in blue (SiR-DNA dye in HeLa-Kyoto BAC cell line and NucBlue dye in non-transfected HeLa). Images are shown in maximum z-projections. Experiments were performed on the non-tranfected HeLa cell line and HeLa-Kyoto BAC cells stably expressing PRC1-GFP. Related to Figures 3A and 4A.

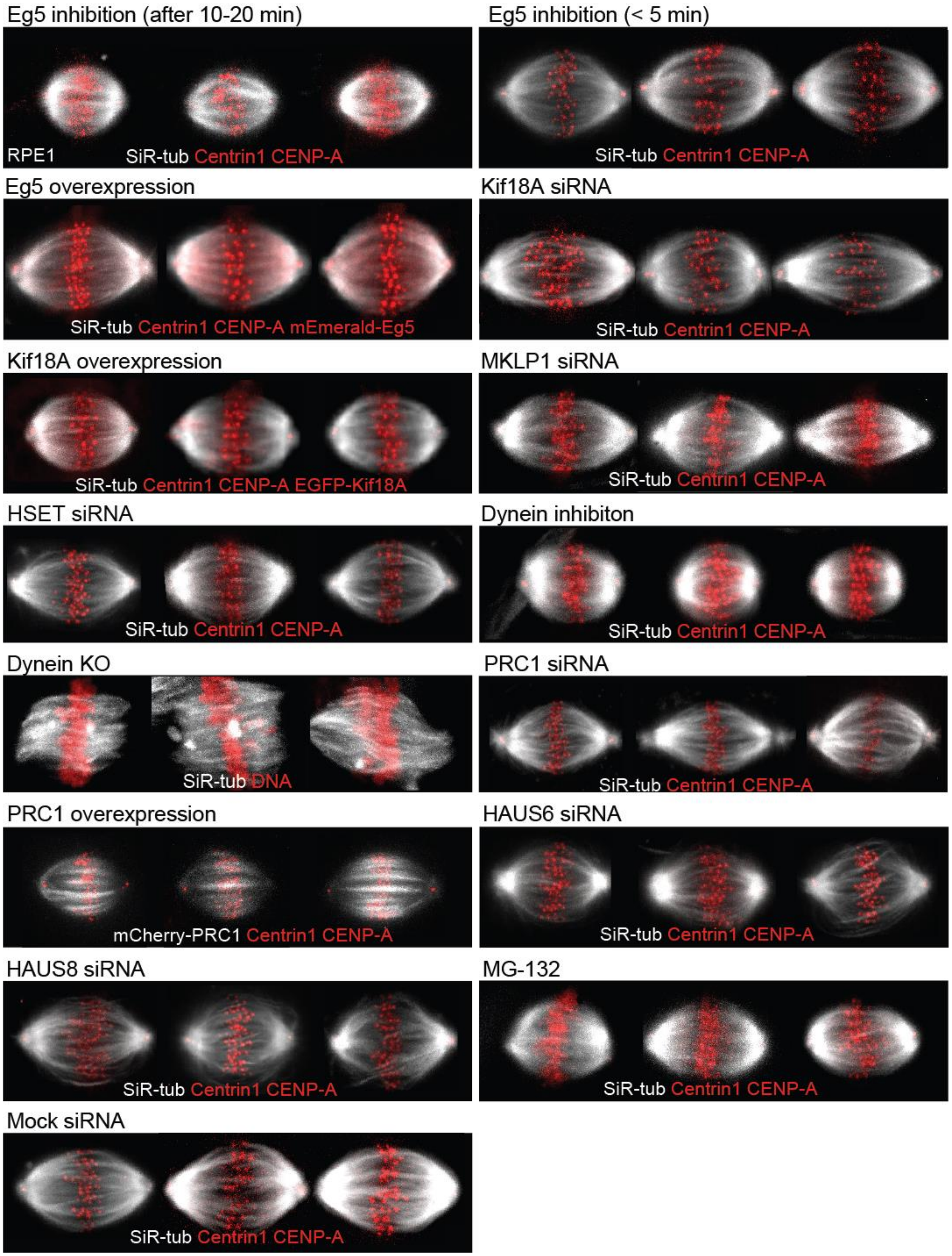
Eg5 inhibition (after 10-20 min)
RPE1
SiR-tub Centrin1 CENP-A
Eg5 inhibition (< 5 min)
SiR-tub Centrin1 CENP-A
Eg5 overexpression
SiR-tub Centrin1 CENP-A mEmerald-Eg5
Kif18A siRNA
SiR-tub Centrin1 CENP-A
Kif18A overexpression
SiR-tub Centrin1 CENP-A EGFP-Kif18A
MKLP1 siRNA
SiR-tub Centrin1 CENP-A
HSET siRNA
SiR-tub Centrin1 CENP-A
Dynein inhibiton
SiR-tub Centrin1 CENP-A
Dynein KO
SiR-tub DNA
PRC1 siRNA
SiR-tub Centrin1 CENP-A
PRC1 overexpression
mCherry-PRC1 Centrin1 CENP-A
HAUS6 siRNA
SiR-tub Centrin1 CENP-A
HAUS8 siRNA
SiR-tub Centrin1 CENP-A
MG-132
SiR-tub Centrin1 CENP-A
Mock siRNA
SiR-tub Centrin1 CENP-A

Figure S5. Spindles of RPE1 cells after perturbation of spindle-associated proteins. Related to Figures 3 and 4.

Examples of three spindles for every perturbation of spindle-associated proteins: Eg5 inhibition (after 10-20 min of STLC treatment), Eg5 inhibition (STLC treatment shorter than 5 min), Eg5 overexpression, Kif18A depletion, Kif18A overexpression, MKLP1 depletion, HSET depletion, dynein inhibition, dynein KO, PRC1 depletion, PRC1 overexpression, HAUS6 and HAUS8 depletion, MG-132 treatment, and mock control. Data was used in the graphs in Figures 3C and 4C. Microtubule bundles are shown in grey (SiR-tubulin and, for PRC1 overexpression, mCherry-PRC1) and kinetochores/centrosomes, DNA (NucBlue dye), Eg5 and KiF18A in red. Images are shown in maximum z-projections. Experiments were performed on hTERT-RPE1 cells, permanently transfected and stabilized using CENP-A-GFP and centrin1-GFP and RPE1 inducible CRISPR/Cas9 DYNC1H1 knockout cells. Related to Figures 3A and 4A.

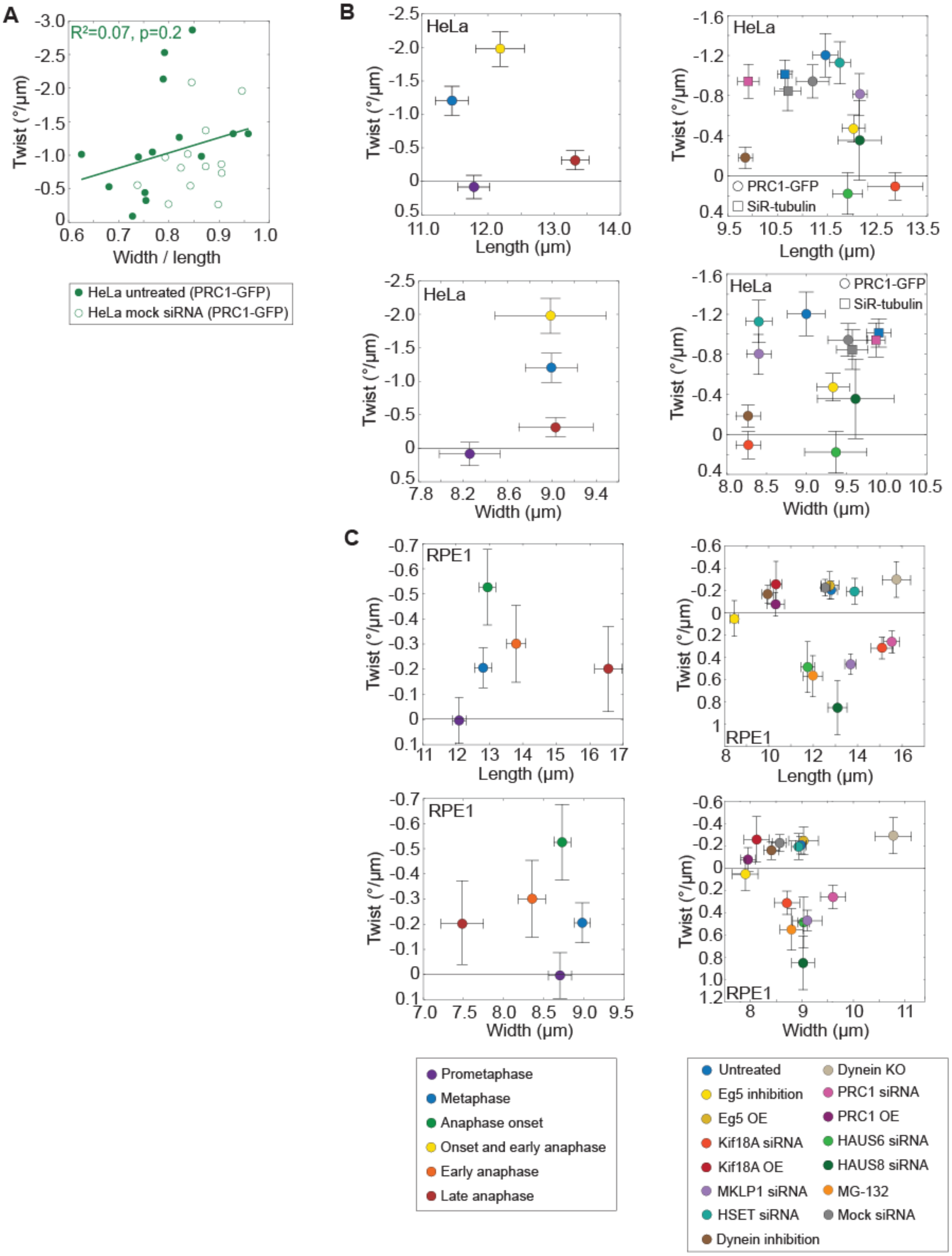
A
R²=0.07, p=0.2
Twist (°/μm)
Width / length
HeLa untreated (PRC1-GFP)
HeLa mock siRNA (PRC1-GFP)
B
HeLa
Twist (°/μm)
Length (μm)
Width (μm)
PRC1-GFP
SiR-tubulin
C
RPE1
Prometaphase
Metaphase
Anaphase onset
Onset and early anaphase
Early anaphase
Late anaphase
Untreated
Eg5 inhibition
Eg5 OE
Kif18A siRNA
Kif18A OE
MKLP1 siRNA
HSET siRNA
Dynein inhibition
Dynein KO
PRC1 siRNA
PRC1 OE
HAUS6 siRNA
HAUS8 siRNA
MG-132
Mock siRNA

Figure S6. Correlation between spindle length or width and spindle twist in different phases of mitosis and during different protein perturbations in HeLa and RPE1 cells. Related to Figure 5.

(A) Round spindles have stronger twist than elongated spindles. Graph shows the correlation between width/length ratio and twist in HeLa cells. Filled circles represent untreated cells while empty circles represent mock siRNA controls. Lines show linear fit (untreated cells together with mock siRNA controls); equation y=-2.28x+0.79; goodness of fit shown in the graph. Same data was used in Figures. 1D, 3B and 4B. Experiments were performed on the HeLa-Kyoto BAC cells stably expressing PRC1-GFP. Related to Figure 5A.

(B) Graphs showing how the spindle twist and length (top) or width (bottom) in HeLa cells vary depending on the different phases of mitosis (left) or perturbations of spindle-associated proteins (right). Each color represents different phase of mitosis or protein perturbation, as described in the legend at the bottom; error bars represent SEM. Same data was used in graphs in Figures. 1D, 3B and 4B. Experiments were performed on the HeLa-Kyoto BAC cells stably expressing PRC1-GFP (dots) and non-tranfected HeLa cell line for the depletion of PRC1 and its controls (rectangles). Related to Figures 5B and 5C.

(C) Graphs showing how the spindle twist and length (top) or width (bottom) in RPE1 cells vary depending on the different phases of mitosis (left) or perturbations of spindle-associated proteins (right). Each color represents different phase of mitosis or protein perturbation, as described in the legend at the bottom; error bars represent SEM. Same data was used in graphs in Figures 1E, 3C and 4C. Experiments were performed on hTERT-RPE1 cells, permanently transfected and stabilized using CENP-A-GFP and centrin1-GFP and RPE1 inducible CRISPR/Cas9 DYNC1H1 knockout cells. Related to Figures 5B and 5C.

SUPPLEMENTAL REFERENCE

S1. Novak, M., Polak, B., Simunic, J., Boban, Z., Kuzmic, B., Thomae, A.W., Tolic, I.M., and Pavin, N. (2018). The mitotic spindle is chiral due to torques within microtubule bundles. Nat Commun 9, 3571.
