# Supplementary material for: The chirality of the mitotic spindle provides a mechanical response to forces and depends on microtubule motors and augmin
Source: Curr Biol. 2022 Jun 6;32(11):2480–2493.e6. doi: 10.1016/j.cub.2022.04.035 (PMC9235856; doi:10.1016/j.cub.2022.04.035)
Supplement: Document S2. Article plus supplemental information [file mmc10.pdf]

Current Biology

Article

The chirality of the mitotic spindle provides a mechanical response to forces and depends on microtubule motors and augmin

Graphical abstract

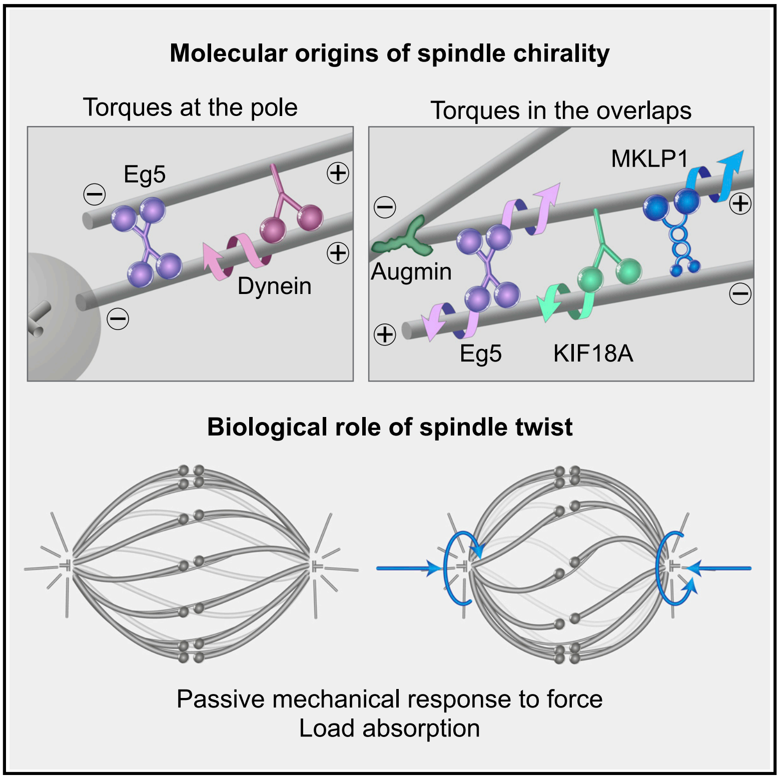

Highlights

- Spindle twist depends on torque-generating motors Eg5, Kif18A, MKLP1, and dynein
- Without the microtubule nucleator augmin, spindles show right-handed twist
- Compression of the spindle along the axis increases the left-handed twist
- Rounder spindles are more twisted than elongated ones are

Authors

Monika Trupinić, Barbara Kokanović, Ivana Ponjavić, Ivan Barišić, Siniša Šegvić, Arian Ivec, Iva M. Tolić

Correspondence

tolic@irb.hr

In brief

Trupinić et al. show that the chirality of the mitotic spindle is most pronounced near the metaphase-to-anaphase transition, and it depends on microtubule motor proteins and augmin. End-on compression of the spindle increases its left-handed twist, suggesting a role of chirality in allowing the spindle to absorb mechanical load.

Trupinić et al., 2022, Current Biology *32*, 2480–2493
June 6, 2022 © 2022 The Author(s). Published by Elsevier Inc.
https://doi.org/10.1016/j.cub.2022.04.035

CellPress

CellPress
OPEN ACCESS

Current Biology

Article

The chirality of the mitotic spindle provides a mechanical response to forces and depends on microtubule motors and augmin

Monika Trupinić,[1] Barbara Kokanović,[1] Ivana Ponjavić,[1] Ivan Barišić,[2] Siniša Šegvić,[2] Arian Ivec,[3] and Iva M. Tolić[1,4,5,*]
[1]Division of Molecular Biology, Laboratory of Cell Biophysics, Ruđer Bošković Institute, Bijenička cesta 54, Zagreb 10000, Croatia
[2]Department of Electronics, Microelectronics, Computer and Intelligent Systems, Faculty of Electrical Engineering and Computing, University of Zagreb, Unska 3, Zagreb 10000, Croatia
[3]Department of Physics, Faculty of Science, University of Zagreb, Bijenička cesta 32, Zagreb 10000, Croatia
[4]Twitter: @Tolicab
[5]Lead contact
*Correspondence: tolic@irb.hr
https://doi.org/10.1016/j.cub.2022.04.035

SUMMARY

Forces produced by motor proteins and microtubule dynamics within the mitotic spindle are crucial for proper chromosome segregation. In addition to linear forces, rotational forces or torques are present in the spindle, which are reflected in the left-handed twisted shapes of microtubule bundles that make the spindle chiral. However, the biological role and molecular origins of spindle chirality are unknown. By developing methods for measuring the spindle twist, we show that spindles are most chiral near the metaphase-to-anaphase transition. To assess the role of chirality in spindle mechanics, we compressed the spindles along their axis. This resulted in a stronger left-handed twist, suggesting that the twisted shape allows for a mechanical response to forces. Inhibition or depletion of motor proteins that perform chiral stepping, Eg5/kinesin-5, Kif18A/kinesin-8, MKLP1/kinesin-6, and dynein, decreased the left-handed twist or led to right-handed twist, implying that these motors regulate the twist by rotating microtubules within their antiparallel overlaps or at the spindle pole. A right-handed twist was also observed after the depletion of the microtubule nucleator augmin, indicating its contribution to the twist through the nucleation of antiparallel bridging microtubules. The uncovered switch from left-handed to right-handed twist reveals the existence of competing mechanisms that promote twisting in opposite directions. As round spindles are more twisted than the elongated ones are, we infer that bending and twisting moments are generated by similar molecular mechanisms and propose a physiological role for spindle chirality in allowing the spindle to absorb mechanical load.

INTRODUCTION

Mitosis is a stage of the cell cycle in which replicated chromosomes are separated into two new nuclei destined for the two daughter cells.[1] In order to segregate the genetic material, the cell forms a mitotic spindle, a complex microstructure made of microtubules and numerous associated proteins.[2–4] The spindle physically separates the chromosomes to the opposite poles of the cell and ensures that each daughter cell has the same number of chromosomes as the parental cell.

The spindle is a mechanical structure that can generate and balance forces within itself.[5] The forces in the spindle are crucial for proper spindle functioning in each phase of mitosis. Kinetochore fibers exert forces necessary for the positioning of the chromosomes at the center of the spindle in metaphase[6–8] and for pulling the chromosomes apart during anaphase.[9–11] On the other hand, overlap bundles balance the forces at kinetochores by acting as bridges between sister kinetochore fibers in metaphase and anaphase[12–15] and also regulate pole separation in anaphase.[16–18] All the forces in the spindle arise from active processes of motor proteins as well as from microtubule polymerization and depolymerization.[19–21] Direct measurement of the forces in the spindle, although possible,[22] is challenging because of the small scales involved.

Forces are also responsible for the shape of a spindle. Because of the mechanical properties of microtubules, which are thin and elastic filaments that are inherently straight and curve under forces, the spindle obtains its characteristic shape.[12,23,24] This means that the spindle shape reflects the forces within it, which allows for an indirect measurement of forces by inferring them from the shapes of the microtubule bundles,[12,25] similarly to studies of forces and shapes of individual microtubules *in vitro*.[26,27]

Recently, it was shown that the shape of the mitotic spindle in human HeLa and U2OS cells is chiral, as the spindle has a left-handed twist around the pole-to-pole axis.[28] Microtubule bundles twist because of the torques that exist within them in addition to linear forces. The experimentally measured three-dimensional shapes of the microtubule bundles, which are primarily bridging fibers that laterally link sister kinetochore fibers and are marked by protein regulator of cytokinesis 1 (PRC1), were used to deduce forces and torques in the spindle by

 © 2022 The Author(s). Published by Elsevier Inc.
This is an open access article under the CC BY license (http://creativecommons.org/licenses/by/4.0/).

CellPress
OPEN ACCESS

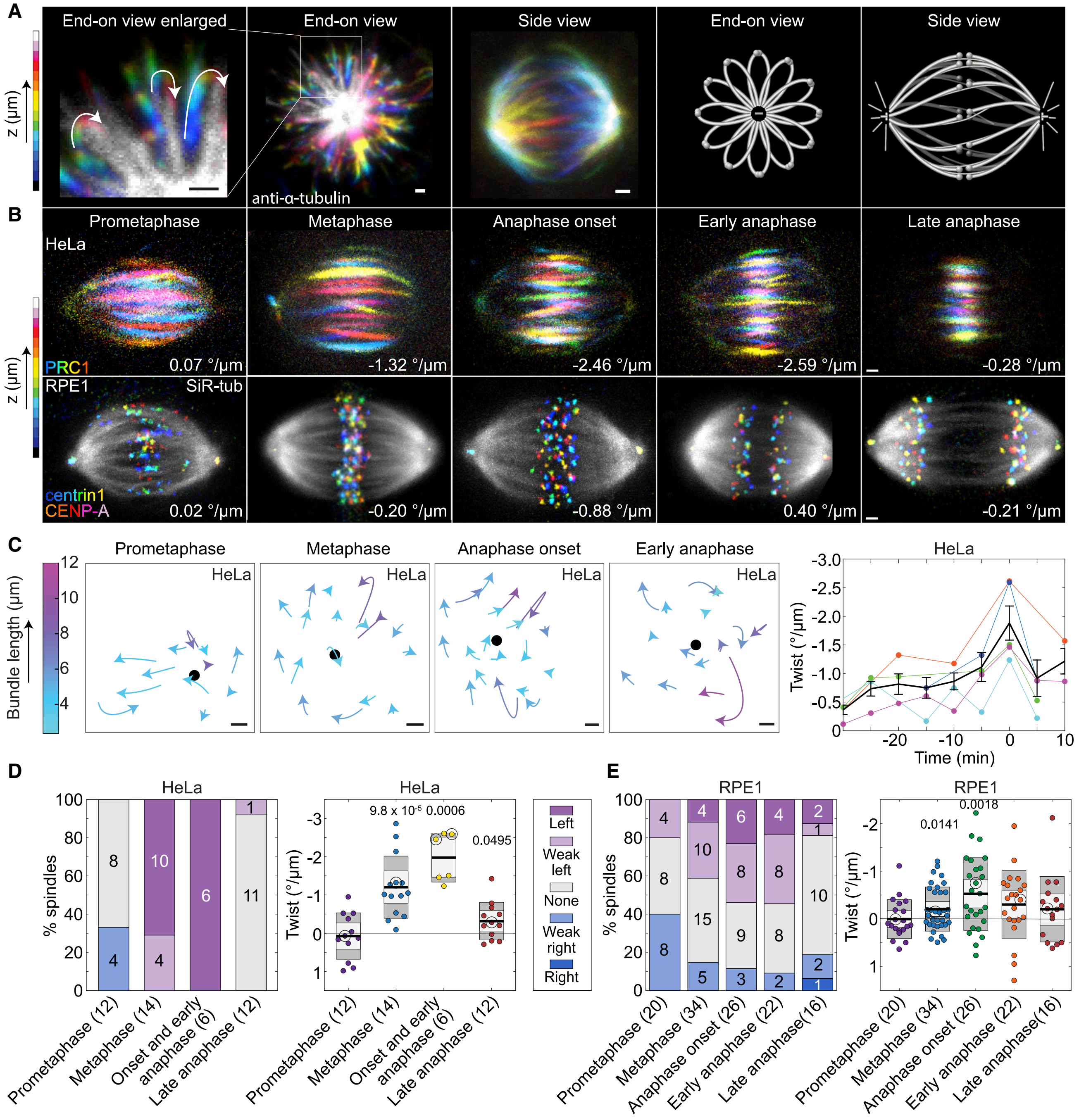

Figure 1. Spindle twist culminates at the beginning of the anaphase

(A) Images of spindles immunostained for α-tubulin in a HeLa-Kyoto BAC cell line stably expressing PRC1-GFP (PRC1-GFP signal is not shown). From left to right: enlarged section of the spindle imaged end-on showing microtubule bundles rotating in a clockwise direction (arrows) through z planes when moving toward the observer, the end-on view and side view of a spindle, and the corresponding schemes. Images are color-coded for depth (see color bar). See also Figure S1A; Video S1.

(B) Top row, the spindles in a HeLa cell line as in (A) in different phases of mitosis. PRC1-GFP signal is color-coded for depth (see the color bar, every second plane was used). Bottom row, the spindles in hTERT-RPE1 cells expressing CENP-A-GFP and centrin1-GFP in different phases of mitosis. Microtubules are shown in gray (SiR-tubulin), and kinetochores/centrosomes are color-coded for depth (color bar) and filtered with a Gaussian blur (radius 0.7). Twist values are given. Additional examples of HeLa cell spindles are shown in Figures S1C and S1D–S1F. See also Videos S2 and S3.

(C) Plots on the left, the microtubule bundle rotation along the z axis from the spindle in a HeLa cell viewed end-on in different phases of mitosis (twist of this spindle is shown in orange in the plot at the right and images in Figure S1C); each microtubule bundle is represented by a circular arc of the circle fitted to its trace with the arrowhead pointing in the rotation direction; the colors denote the bundle length along the pole-to-pole axis (see color bar); the black dot represents the

(legend continued on next page)

comparison with a theoretical model.[28] A left-handed twist was also observed in spindles lacking NuMA and kinesin-5 activity in RPE1 cells during anaphase.[29] Another organism whose spindles are prominently twisted is a unicellular eukaryote, amoeba *Naegleria gruberi*. Interestingly, the amoeba's spindles are predominantly twisted in a right-handed fashion.[30]

The twist of the spindle is potentially generated by motor proteins that, in addition to linear forces, exert rotational forces on microtubules by switching protofilaments with a bias in a certain direction.[31–42] The first molecular motor discovered to generate torque was the single-headed axonemal dynein. In *in vitro* gliding motility assays, surface-attached dynein motors rotated the microtubules around their axis in a clockwise motion, when viewed from the minus-end of the microtubules while translocating them in a linear fashion.[31] Similar microtubule rotation was observed with the minus-end-directed motor kinesin-14 (Ncd).[32,41] Counterclockwise rotation was found for the plus-end-directed motor kinesin-5 (Eg5)[34] and kinesin-8 (Kip3),[36,40] whereas another study found that kinesin-8 can switch protofilaments in both directions.[38] Several other motor proteins also exhibit rotational movements, including kinesin-1,[33,39] kinesin-2,[35] cytoplasmic dynein,[37] and kinesin-6 (MKLP1).[42] However, in contrast to this large body of knowledge on chiral motor stepping *in vitro*, the role of motor proteins and their asymmetric stepping in the generation of torques within microtubule bundles *in vivo* and consequently spindle twist are unknown.

In this paper, we address the biological role and the molecular origin of spindle chirality. We show that spindle twist changes through the different phases of mitosis and peaks around anaphase onset. To test the idea that the chiral shape may help the spindle to absorb mechanical load, we compressed the spindles along the pole-to-pole axis, which led to an increase in spindle twist. Thus, we propose a biological function of spindle chirality in promoting the flexibility of the spindle and its mechanical response to external forces. By performing a candidate screen in which we depleted or inactivated motor proteins that step in a chiral manner and other microtubule-associated proteins, we identified several molecular players involved in the regulation of spindle chirality, leading us to suggest that the main mechanism generating spindle chirality is the action of motor proteins that rotate microtubules around one another within the antiparallel overlaps.

RESULTS

Spindle twist is most pronounced at anaphase onset in a cancer cell line and a non-cancer cell line

To explore the twist of the spindle, the first step was to obtain end-on view images covering the whole spindle from pole to pole because this view allows for the visualization of the twist of microtubule bundles (Figure 1A [end-on view]; Video S1). A signature of the twisted shape is that microtubule bundles look like flower petals in the end-on view. In contrast, the twisted shape is not easily recognized in the side view of the spindle (Figure 1A [side view]).

To quantify spindle twist, we used the following three complementary approaches: visual assessment, optical flow, and bundle tracing (Figure S1A; STAR Methods). As it is still an open question in the field as to what method is the most appropriate to measure spindle twist,[28,29,43] visual assessment is useful as a quick and rough estimate of the twist and as a control for automated or semi-automated methods. In this method, the spindle is observed end-on and the rotation of the microtubule bundles around the pole-to-pole axis is estimated visually. If the bundles rotate clockwise when moving along the spindle axis in the direction toward the observer, the twist is left-handed, and vice versa (Figure S1A [left]). We score the twist as left-handed, right-handed, weak left-handed, weak right-handed, or no visible twist. Weak twists correspond to a range of $\sim-1°/\mu m$ to $-2°/\mu m$ and left and right twists to stronger rotations (Figure S1B; STAR Methods). In the optical flow method, the movement of the signal coming from the microtubule bundles is estimated by comparing the signal from one z plane to the next (Figure S1A [middle]; STAR Methods). This method provides a value for the average twist of all bundles in a spindle, and it is optimal for experiments on a large number of spindles because it is automated. The bundle tracing method is an extension of the approach developed previously,[28] where individual bundles are manually traced by following the bundle contour in the end-on view of the spindle (Figure S1A [right]; STAR Methods). Subsequently, a circle is fitted to the bundle trace and used to calculate the curvature and twist of individual bundles[43] (Figure S1A [right]; STAR Methods).

As a label for microtubule bundles, we used SiR-tubulin to observe all microtubule bundles or PRC1-GFP to observe the bridging fibers.[12,13] To compare the results of the three methods, we analyzed the twist of 10 metaphase spindles in HeLa cells stably expressing PRC1-GFP (Figure S1B). All three methods yielded a left-handed twist, which is expressed by negative values. The absolute values of the twist of individual spindles obtained by bundle tracing and optical flow were similar, with optical flow yielding smaller negative values ($-1.32°/\mu m \pm 0.29°/\mu m$, n = 10; all data are given as mean ± SEM) than bundle tracing ($-2.07°/\mu m \pm 0.29°/\mu m$, n = 10). This difference is likely due to the sensitivity of the optical flow method to all signals, including the background. Based on this cross-check between the three methods, we conclude that they provide comparable values of spindle twist. Thus, we use optical flow for experiments in which

pole-to-pole axis. The graph on the right shows spindle twist in five HeLa cells over time; the beginning of anaphase (visible chromosome separation) was set as time zero; each color represents one cell; the black line with error bars represents mean ± SEM.

(D) Twist in different phases of mitosis in HeLa-Kyoto BAC cells expressing PRC1-GFP. Left, visual assessment graph represents the percentages of spindles showing left, right, weak left, weak right, or no twist (see legend); numbers in the bars and in brackets show the number of cells. Right, the twist values calculated with the optical flow method. The black line shows the mean; the light and dark gray areas mark 95% confidence interval on the mean and standard deviation, respectively; numbers above the data show p values (Student's t test for the mean twist value different from 0). Non-significant differences are not shown. The circled dots represent the cells that are shown in the images above. Raw data of 10 out of 14 metaphase spindles were re-calculated from Novak et al.[28] and also used in Figure S1B. See also Figure S1G.

(E) Twist in different phases of mitosis in hTERT-RPE1 cells expressing CENP-A-GFP and centrin1-GFP; legend as in (D). All scale bars, 1 μm.

CellPress
OPEN ACCESS

we test changes in the overall spindle twist in a large number of cells and bundle tracing for experiments where high spatial precision is required.

Spindles in cancer cell lines are twisted in a left-handed manner in metaphase,[28] but it is not known whether the twist is present already when the spindle assembles in prometaphase or whether it arises as the spindle matures. Furthermore, it is unknown how the twist changes during anaphase. To examine the development of spindle twist throughout mitosis (Figure 1B; Videos S2 and S3), we first measured the twist in individual live HeLa cells expressing PRC1-GFP as they progressed through mitosis (Figures 1C and S1C). The average twist of the spindle in prometaphase was close to 0; it was left-handed (negative) during metaphase, culminated at anaphase onset reaching a value of $-1.88°/\mu m \pm 0.3°/\mu m$ (n = 5), and decreased afterward (Figure 1C). In agreement with this result, experiments in which different spindles were imaged in different phases showed a peak of spindle twist around anaphase onset, with a value of $-1.98°/\mu m \pm 0.26°/\mu m$ (n = 6) (Figures 1B, 1D, and S1D–S1F; Videos S2 and S3; Table 1). Expression of PRC1-GFP in this cell line did not influence the twist, as non-transfected HeLa cells stained with SiR-tubulin showed similar twist values in metaphase (Table 1; p = 0.47, Student's t test).

To test whether the time spent in metaphase affects spindle twist, we accelerated entry into anaphase by inhibiting Mps1 kinase, one of the main components of the spindle assembly checkpoint.[44] The treatment of HeLa cells expressing PRC1-GFP with the inhibitor AZ3146[45] during prometaphase shortened the time to anaphase from 40 min to 8–10 min on average. We measured the twist at the beginning of anaphase or in early anaphase and found it to be significantly smaller than in control cells, $-0.17°/\mu m \pm 0.21°/\mu m$ (n = 17) (Figure S1G; Table 1). This result suggests that the reduction of time needed to enter the anaphase may also mean a reduction of time to build up the spindle twist.

To explore whether spindle twist and its variation over time are specific to cancer cell lines, we measured the twist in the non-cancer immortalized epithelial cell line hTERT-RPE1 (from here on referred to as RPE1) (Figure 1B) and found that spindles in these cells also showed a left-handed twist, but the values were smaller than in HeLa cells (Figure 1E). Moreover, the temporal pattern of twist in RPE1 cells was similar to that in HeLa cells. Twist was absent in prometaphase; it was very weak left-handed in metaphase and was at its peak value at anaphase onset; it decreased during anaphase and vanished in late anaphase (Figure 1E; Videos S2 and S3; Table 1). The value at anaphase onset was $-0.53°/\mu m \pm 0.15°/\mu m$ (n = 26), which indicates a weaker left-handed twist than in HeLa cells. Taken together, our results show that spindles are born without a twist. The left-handed twist in HeLa cells arises as the spindle acquires its metaphase shape, peaks at the start of chromosome segregation, and declines afterward. In RPE1 cells, the twist shows a similar trend, but the values are much less pronounced, and the twist is mostly noticeable only in early anaphase.

Compression of the spindle along the pole-to-pole axis increases the left-handed twist

The biological role of spindle chirality is still unknown. Although chirality may be simply a side effect of the activity of torque-generating motors, the twisted shapes of microtubule bundles may contribute to spindle physiology by allowing changes of spindle shape as a mechanical response to external forces. To test this idea, we gently compressed vertically oriented HeLa cell spindles in metaphase along the pole-to-pole axis for 1.5 min, following the compression protocol from a previous study[46] (Figure 2A; Video S4). We used the bundle tracing method to measure spindle twist, which allowed us to graphically reconstruct spindles from the end-on view and side view (Figure 2B). Traces of the microtubule bundles in the end-on view after 1 min of compression were more rounded than before compression, indicating an increase in twist, and the mitotic spindle shortened (Figure 2B). Spindle shortening was used as a measure to confirm successful compression, showing that spindle length decreased from $14.07 \pm 0.55\ \mu m$ before compression to $12.75 \pm 0.80\ \mu m$ after 1 min of compression (p = 0.013; a paired t test was used to compare the values before and after compression, n = 6 spindles) (Figure 2C). Spindle width increased after compression in some cases, e.g., for the spindle shown in Figure 2B, but overall, this change was not significant (p = 0.18) (Figure 2D).

Interestingly, compression resulted in a 2.3-fold increase of the left-handed spindle twist, from $-0.63°/\mu m \pm 0.28°/\mu m$ before compression to $-1.42°/\mu m \pm 0.50°/\mu m$ after 1 min of compression (p = 0.040) (Figures 2E and S2A; Video S4). Histograms of twist values show that the distribution shifted toward more negative values upon compression (Figure S2B). To quantify this shift, we analyzed the fraction of bundles having a strong left-handed twist with a value smaller than $-2.8°/\mu m$, which is one standard deviation away from the mean twist before compression. The twist was smaller than $-2.8°/\mu m$ for 9 out of 80 bundles (11.3% ± 3.5%) before compression, whereas after compression this was the case for 21 out of 73 bundles (28.8% ± 5.3%). The difference was significant (p = 0.0064; two-proportions z test), suggesting that the compression resulted in a higher proportion of bundles having a strong left-handed twist.

Contour length of the microtubule bundles did not change significantly after compression (p = 0.99) (Figures 2F and S2A). We were unable to detect changes in bundle curvature after compression (p = 0.41) (Figures 2G and S2A), which is consistent with the non-significant change in spindle width. Thus, as the spindle was compressed end-on by an external force, which resulted in spindle shortening, the microtubule bundles did not shorten substantially but instead became more twisted. These results support the idea that the twist within the bundles allows for a mechanical response to external forces.

Motor proteins Eg5/kinesin-5, Kif18A/kinesin-8, MKLP1/kinesin-6, and dynein regulate spindle twist

To explore the molecular origins of torques in the spindle and thus its twisted shape, we consider the following molecular activities. First, motors that exert torque on the microtubule may generate the twisted shape of the bundle by twisting the microtubules within the bundle around each other or by twisting the microtubules with respect to the spindle pole. Second, proteins that crosslink neighboring microtubules or link microtubules with the pole may prevent free rotation of the microtubules, thereby allowing for twisting of the bundles. Third, nucleation of new microtubules within the bundle may affect the bundle twist.

Table 1. Spindle twist, length, and width in HeLa and RPE1 cells in different phases of mitosis and after protein perturbations

	HeLa			RPE1		
	Twist (°/μm)	Length (μm)	Width (μm)	Twist (°/μm)	Length (μm)	Width (μm)
Prometaphase	0.09 ± 0.18 (12)	11.8 ± 0.2	8.3 ± 0.3	0.004 ± 0.09 (20)	12.1 ± 0.2	8.7 ± 0.1
Metaphase	−1.20 ± 0.22 (14)	11.5 ± 0.3	9.0 ± 0.2	−0.21 ± 0.08 (34)	12.8 ± 0.3	9.0 ± 0.1
	* −1.01 ± 0.14 (14)	*10.6 ± 0.1	*9.9 ± 0.2			
Anaphase onset	−1.98 ± 0.26 (6)	12.2 ± 0.4	9.0 ± 0.5	−0.53 ± 0.15 (26)	12.9 ± 0.2	8.7 ± 0.1
Early anaphase				−0.30 ± 0.15 (22)	13.8 ± 0.3	8.4 ± 0.2
Late anaphase	−0.31 ± 0.14 (12)	13.3 ± 0.2	9.0 ± 0.3	−0.20 ± 0.17 (16)	16.6 ± 0.4	7.5 ± 0.3
Mps1 inhibition	−0.17 ± 0.21 (17)	11.9 ± 0.4	8.4 ± 0.2	n.d.	n.d.	n.d.
Eg5 inhibition (after < 5 min)	−0.47 ± 0.14 (16)	12.0 ± 0.2	9.3 ± 0.2	−0.06 ± 0.19 (11)	12.3 ± 0.4	8.8 ± 0.1
Eg5 inhibition (after 10–20 min)	n.d.	n.d.	n.d.	0.06 ± 0.13 (12)	8.3 ± 0.2	7.7 ± 0.2
Eg5 overexpression	n.d.	n.d.	n.d.	−0.25 ± 0.12 (11)	12.7 ± 0.4	9.0 ± 0.3
Kif18A siRNA	0.11 ± 0.14 (21)	12.8 ± 0.6	8.3 ± 0.2	0.30 ± 0.11 (24)	15.0 ± 0.6	8.7 ± 0.2
Kif18A overexpression	n.d.	n.d.	n.d.	−0.26 ± 0.20 (7)	10.3 ± 0.3	8.1 ± 0.2
MKLP1 siRNA	−0.80 ± 0.19 (12)	12.1 ± 0.1	8.4 ± 0.2	0.48 ± 0.10 (16)	13.6 ± 0.3	9.1 ± 0.3
HSET siRNA	−1.13 ± 0.21 (17)	11.7 ± 0.2	8.4 ± 0.2	−0.19 ± 0.12 (18)	13.9 ± 0.4	8.9 ± 0.1
Dynein inhibition	−0.18 ± 0.11 (18)	9.8 ± 0.1	8.3 ± 0.2	−0.18 ± 0.08 (16)	9.8 ± 0.3	8.4 ± 0.1
Dynein KO	n.d.	n.d.	n.d.	−0.29 ± 0.13 (15)	15.8 ± 0.8	10.7 ± 0.5
PRC1 siRNA	*−0.94 ± 0.17 (19)	*9.9 ± 0.2	*9.9 ± 0.1	0.22 ± 0.11 (22)	15.3 ± 0.4	9.6 ± 0.2
PRC1 overexpression	n.d.	n.d.	n.d.	−0.08 ± 0.11 (10)	10.3 ± 0.4	8.0 ± 0.1
HAUS6 siRNA	0.18 ± 0.21 (16)	11.9 ± 0.3	9.4 ± 0.4	0.49 ± 0.21 (16)	11.7 ± 0.3	9.0 ± 0.1
HAUS8 siRNA	−0.35 ± 0.40 (10)	12.1 ± 0.4	9.6 ± 0.5	0.85 ± 0.24 (13)	13.1 ± 0.4	9.0 ± 0.2
Mock siRNA	*−0.85 ± 0.20 (17)	*10.7 ± 0.3	*9.6 ± 0.2	−0.22 ± 0.08 (39)	12.5 ± 0.2	8.6 ± 0.1
	−0.94 ± 0.16 (13)	11.2 ± 0.3	9.5 ± 0.3			
MG-132	n.d.	n.d.	n.d.	0.51 ± 0.14	12.0 ± 0.4	8.8 ± 0.2

All values are shown as mean ± SEM. The numbers in the brackets denote the number of cells; n.d., not determined; * represents non-transfected HeLa cells (the rest of the data on HeLa cells comes from HeLa-Kyoto BAC cells stably expressing PRC1-GFP); RPE1 cells used were hTERT-RPE1 cells permanently transfected and stabilized using CENP-A-GFP and centrin1-GFP.

To test the role of these activities in the regulation of spindle twist, we performed a candidate screen on HeLa and RPE1 cells in which we perturbed motor proteins and other microtubule-associated proteins one by one using siRNA-mediated depletion, small-molecule inhibitors, or overexpression and measured the resulting spindle twist. As the candidates for this mini screen, we selected spindle-localized motor proteins for which it has been shown *in vitro* that they can rotate the microtubule (Eg5/kinesin-5, Kif18A/ kinesin-8, MKLP1/kinesin-6, HSET/kinesin-14, and dynein), the main crosslinker of antiparallel microtubules PRC1, and the augmin complex that is responsible for the nucleation of microtubules along existing microtubules. Spindle twist was measured during metaphase, rather than at the anaphase onset when the twist is most pronounced, because depletion or inhibition of some of the candidate proteins, such as Eg5, Kif18A, and augmin, interferes with anaphase entry.[47–49] Furthermore, the measurement of the twist in metaphase is more reproducible because spindles in metaphase are in a steady state, whereas anaphase spindles undergo extensive changes. All candidate proteins were depleted by siRNA, except Eg5 and

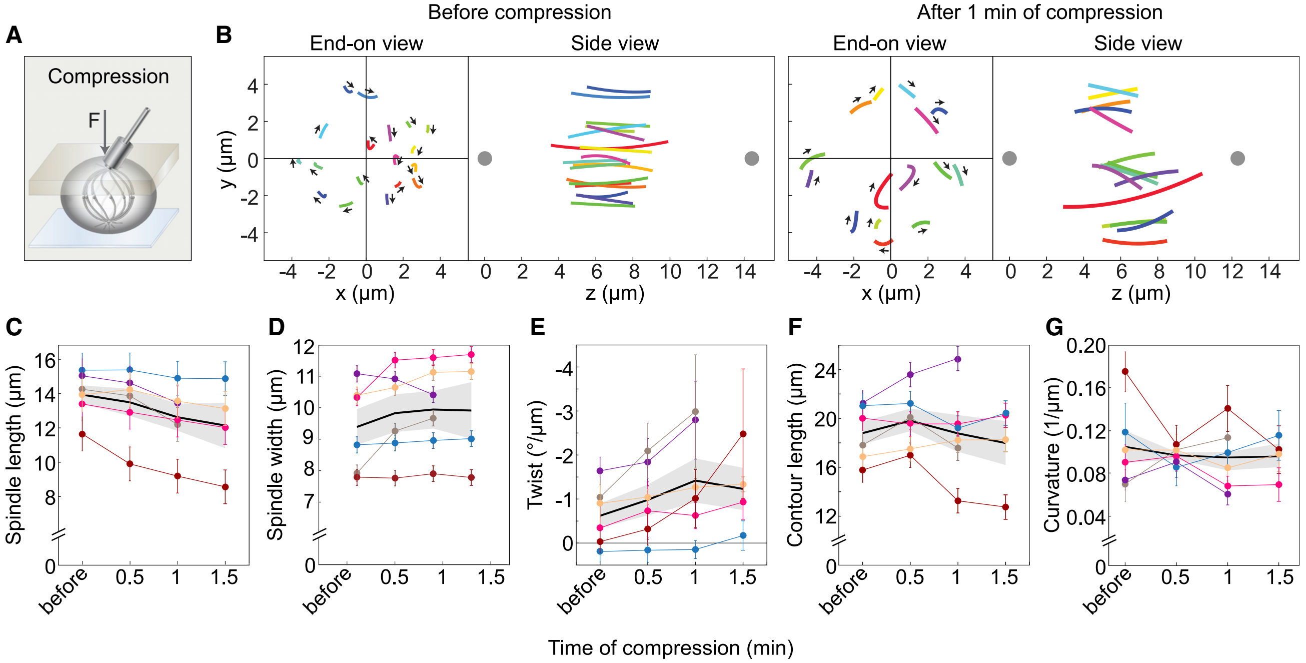

Figure 2. The spindles compressed by an external force have a stronger twist

(A) Setup for spindle compression. The blue layer represents the dish and the gray layer the gel with a metal rod on top; arrow shows the direction of force, *F*.

(B) Microtubule bundles in a spindle shown from the end-on and side view before compression and after 1 min of compression, as indicated. In the end-on and side view, the individual bundles are colored with the same color, but colors before and after compression do not represent the same bundles; the lines show circular arcs of the fitted circles and arrows represent the rotation direction; the gray dots are spindle poles.

(C–G) Spindle parameters from before compression up to 1.5 min of compression are as follows: spindle length (C), spindle width (D), twist of microtubule bundles (E), length of the bundle contours (F), and bundle curvature (G). Each color represents one cell; the dots represent mean values; the error bars in (C) and (D) show the estimated errors in the determination of spindle length and width, 1 and 0.25 µm, respectively; the error bars in other graphs represent SEM. The black line and gray area represent mean ± SEM. Successful compression was performed on six spindles from five independent experiments on HeLa-Kyoto BAC cells expressing PRC1-GFP. Individual data points are shown in Figure S2A. See also Figure S2B; Video S4.

dynein. Eg5 was inhibited with S-trityl-L-cysteine (STLC)[50] because siRNA depletion of Eg5 would not allow for spindles to properly assemble, resulting in monoasters.[49] For dynein inhibition, we used dynarrestin[51] in both HeLa and RPE1 cells, as well as CRISPR/Cas9-inducible DYNC1H1 (dynein heavy chain) knockout (KO) RPE1 cells.[52] Depletion by siRNA of each protein was confirmed by measurements of the immunofluorescence signal of that protein on the spindle (Figures S3A and S3B).

In agreement with our previous work on HeLa cells,[28] we found that the acute inhibition of Eg5 with STLC decreased the left-handed spindle twist in both HeLa and RPE1 cells (Figures 3A, 3B, 3C, S4, and S5; Table 1). In RPE1 cells, the spindles had no twist 5 min after STLC addition, whereas the spindle length was the same as before the treatment and after 10–20 min when the spindles were shorter but still bipolar (Table 1; Figure S5). These results suggest that changes in the spindle twist due to Eg5 inhibition are independent of the changes in spindle length.

Depletion of Kif18A abolished spindle twist in HeLa cells and, intriguingly, resulted in a right-handed twist in RPE1 cells, causing ~71% of RPE1 spindles to twist in the right-handed fashion, with a mean twist of 0.30°/µm ± 0.11°/µm ($n = 24$, $p = 0.0119$ for a difference from 0 in a Student's t test; Figures 3A–3C, S4, and S5; Video S5; Table 1). Overexpression of either Eg5 or Kif18A in RPE1 cells did not yield changes in the twist (Figures 3C and S5; Table 1).

Depletion of MKLP1 did not change the twist in HeLa cells but significantly changed the twist in RPE1 cells, where 94% of spindles were twisted in a right-handed direction (Figures 3A–3C, S4, and S5; Video S6; Table 1). The mean twist was 0.48°/µm ± 0.10°/µm ($n = 16$, $p = 0.0003$ for a difference from 0 in a Student's t test). Depletion of HSET/kinesin-14 did not change the twist (Figures 3A–3C, S4, and S5; Table 1). Dynarrestin treatment abolished the twist in HeLa cells but did not change the twist in RPE1 cells (Figures 3A–3C, S4, and S5; Video S7; Table 1). In DYNC1H1 knockout RPE1 cells, the twist was absent, but it was challenging to measure the twist in these cells due to the unfocused spindle poles and altered spindle shape (Figure 3C and S5; Video S7; Table 1). We conclude that Eg5, Kif18A, MKLP1, and dynein regulate the torques within the spindle, which lead to the twisted shape of microtubule bundles, but their contribution differs in different cell lines.

Depletion or overexpression of PRC1 in RPE1 spindles results in no twist

PRC1 protein is not only a key regulator of cytokinesis[53] but also the main crosslinking protein of antiparallel microtubules within bridging fibers.[12,13] Without PRC1, bridging fibers are thinner and spindles have a less curved and more diamond-like shape,[12,54] which led us to hypothesize that the twist might also be affected. In HeLa cells, depletion of PRC1 did not yield

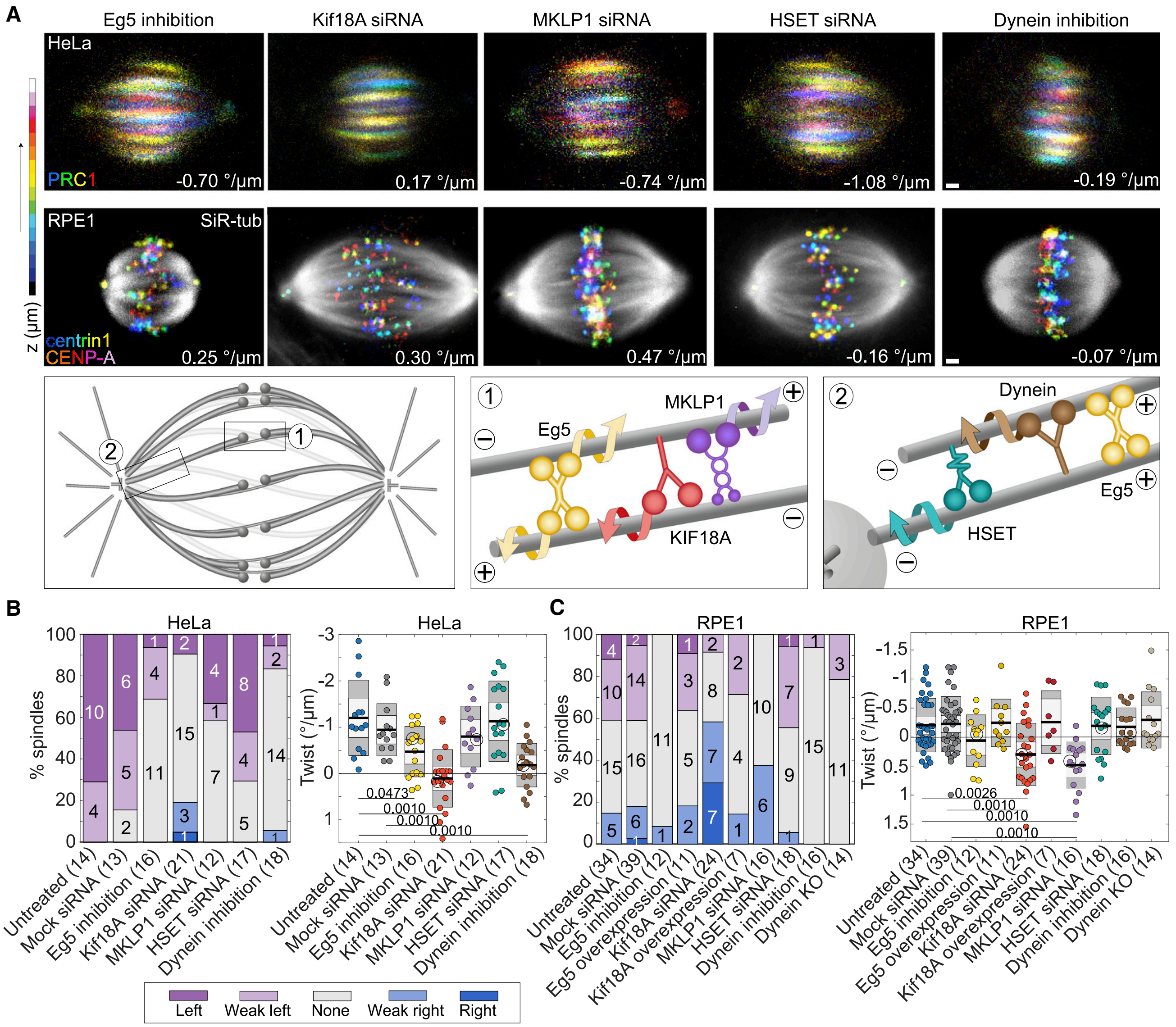

Figure 3. Motor proteins Eg5 and Kif18A control spindle twist

(A) First row, spindles in HeLa-Kyoto BAC cells expressing PRC1-GFP after inhibition/depletion of Eg5, Kif18A, MKLP1, HSET, and dynein. Second row, spindles in hTERT-RPE1 cells expressing CENP-A-GFP and centrin1-GFP after the same inhibitions/depletions, as indicated. Color-coding for depth as in Figure 1B. Scale bars, 1 μm. Additional examples are shown in Figures S4 and S5. Third row, schemes showing localization and movement of the targeted motor proteins within the spindle. See also Videos S5, S6, and S7.

(B) Spindle twist after perturbations of motor proteins in HeLa cells expressing PRC1-GFP. Left, visual assessment of twist; right, twist calculated with the optical flow method; legend as in Figure 1D. On the right, one-way ANOVA test showed a significant difference between group means ($p = 1.39 \times 10^{-8}$); numbers below the data show p values (Tukey's HSD post hoc test); non-significant differences are not shown; the encircled dots represent cells on the images above. The immunofluorescence of the targeted proteins after perturbations is shown in Figure S3A.

(C) Spindle twist after perturbations of motor proteins in RPE1 cells expressing CENP-A-GFP and centrin1-GFP and RPE1 inducible CRISPR/Cas9 DYNC1H1 knockout cells, legend as in (B). One-way ANOVA test showed a significant difference between group means ($p = 2.27 \times 10^{-7}$). Immunofluorescence after perturbations is shown in Figure S3B. Data for Eg5 inhibition correspond to 10–20 min after STLC addition.

changes in the spindle twist (Figures 4A, 4B, and S4; Table 1). When we depleted PRC1 in RPE1 cells, the spindles had no twist on average (Figures 4A, 4C, and S5; Table 1). Overexpression of PRC1 in RPE1 cells also resulted in the abolishment of the spindle twist, as the microtubule bundles became almost straight (Figures 4A, 4C, and S5; Table 1). These data suggest that PRC1 regulates torques within the spindle in RPE1 cells, possibly by limiting the free rotation of microtubules within antiparallel bundles and by modulating the torsional rigidity of the bundle.

Depletion of augmin leads to no twist in HeLa cells and right-handed twist in RPE1 cells

The augmin complex is responsible for the microtubule nucleation from the lateral surface of the pre-existing microtubules.[48,55]

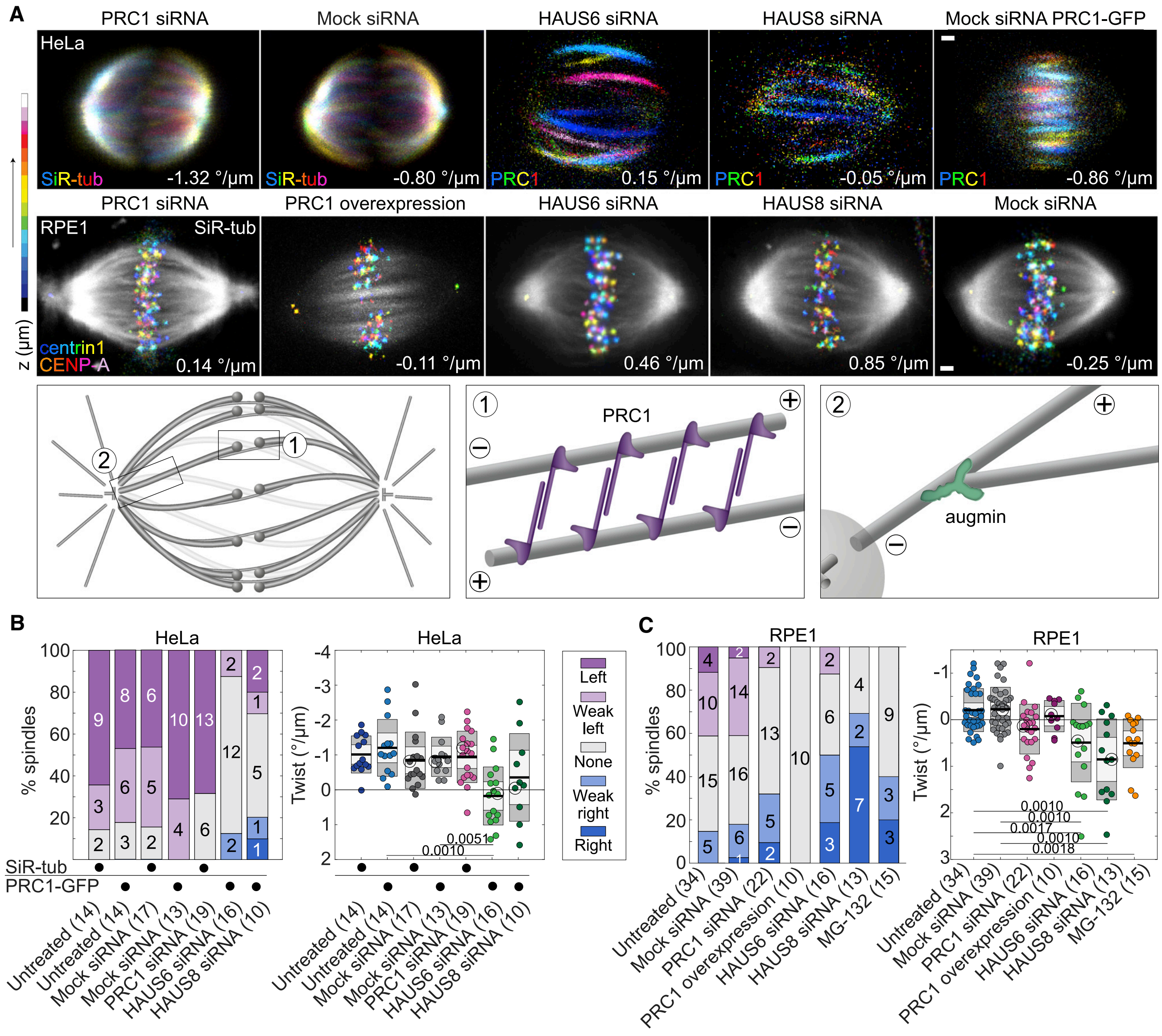

Figure 4. Microtubule crosslinker PRC1 and nucleator augmin regulate spindle twist

(A) First row, spindles in a non-transfected HeLa cell line stained with SiR-tubulin (first 2 spindles) and HeLa-Kyoto BAC cells expressing PRC1-GFP (last 3 spindles), after depletion of PRC1 and subunits of the augmin complex HAUS6 or HAUS8. Second row, images of spindles in hTERT-RPE1 cells expressing CENP-A-GFP and centrin1-GFP after perturbations of PRC1 and depletions of HAUS6 or HAUS8, as indicated. Color-coding for depth as in Figure 1B (see color bar), except SiR-tubulin is shown in non-transfected HeLa cells. In RPE1 spindles, gray represents SiR-tubulin, except in the cell with overexpressed PRC1 that shows PRC1-mCherry. Scale bars, 1 μm. Additional examples are given in Figures S4 and S5. Third row, the schemes showing the localization of PRC1 and augmin in the spindle. See also Video S8.

(B) Spindle twist after perturbations of PRC1 and augmin in HeLa cells. Left, visual assessment of twist; right, twist calculated with the optical flow method; legend as in Figure 1D. The black dots denote the cell line/staining used for the corresponding treatment. On the right, the one-way ANOVA test showed a significant difference between group means ($p = 8.06 \times 10^{-5}$); numbers below the data show p values (Tukey's HSD post hoc test); non-significant differences are not shown; the encircled dots represent cells shown on images. The experiments were performed on non-transfected HeLa cells (for the depletion of PRC1 and its control) and HeLa-Kyoto BAC cells expressing PRC1-GFP (for the depletion of HAUS6 and HAUS8 and their controls). Immunofluorescence after protein perturbations is shown in Figure S3A.

(C) Spindle twist after perturbations of PRC1 and augmin in RPE1 cells expressing CENP-A-GFP and centrin1-GFP; legend as in (B). One-way ANOVA test showed a significant difference between group means ($p = 1.72 \times 10^{-9}$); immunofluorescence is shown in Figure S3B.

Augmin is important for the nucleation of the bridging fibers and, consequentially, the maintenance of the spindle shape.[56] When we depleted the augmin subunit HAUS6 (hDgt6/FAM29A), which binds to γTuRC through the adaptor protein NEDD1,[48] the spindles in the HeLa cells had zero twist on average, whereas those in RPE1 cells had a right-handed twist of 0.49°/μm ± 0.21°/μm ($n = 16$, $p = 0.0341$ for a difference from 0 in a Student's t test; Figures 4A–4C, S4, and S5; Table 1).

A similar result was observed after the depletion of the augmin subunit HAUS8 (hDgt4/Hice1), which binds to pre-existing microtubules.[57,58] This resulted in zero average twist in HeLa cells and a strong right-handed twist in RPE1 cells of 0.85°/μm ± 0.24°/μm (n = 13, p = 0.0041 for a difference from 0 in a Student's t test; Figures 4A–4C, S4, and S5; Video S8; Table 1). The twist after the depletion of HAUS6 or after the depletion of HAUS8 was not significantly different in HeLa (p = 0.26) or RPE1 cells (p = 0.27) as expected, given that they are part of the same complex. Thus, the augmin-mediated nucleation of microtubules along the wall of pre-existing microtubules is an important determinant of the direction and amount of spindle twist.

As depletion of the augmin complex subunits prolongs metaphase,[48] we explored how the twist changes when cells are arrested in metaphase by adding the proteasome inhibitor MG-132. Interestingly, the spindles in RPE1 cells that arrested in metaphase had a right-handed twist of 0.51°/μm ± 0.14°/μm (n = 15, Figure 4C and S5; Table 1), suggesting that prolonging metaphase may cause a shift in the balance of torque-generating activities resulting in a right-handed twist.

Round spindles are more twisted than elongated spindles

To explore the relationship between twisting and bending moments in the spindle, we tested the correlation between spindle twist and width/length ratio, as higher aspect ratios are a signature of stronger bending moments in the spindle.[28] In non-transfected HeLa cells, whose width/length ratios were roughly between 0.8 and 1, rounder spindles had a stronger left-handed twist (Figure 5A), indicating a correlation between bending and twisting moments. In contrast, no correlation was observed in RPE1 cells, whose width/length ratios were between 0.5 and 0.8 (Figure 5A). A weak correlation was found in HeLa cells expressing PRC1-GFP, which had smaller width/length ratios than non-transfected HeLa cells (Figure S6A).

A plot of the twist as a function of the width/length ratio for various mitotic phases and treatments indicates that different combinations of twist and bending moments exist in spindles in different phases of mitosis or in which different molecular mechanisms are perturbed (Figures 5B and 5C; see Figure S6 for twist versus width or length). In HeLa cells, prometaphase and late anaphase spindles are elongated with zero and small left-handed twist values, respectively (Figure 5B). Left-handed twist rises during metaphase when spindles are the roundest, and the highest twist values are at the beginning of anaphase when spindles are still rather round (Figure 5B). In contrast, in RPE1 cells, such a correlation between twist and roundness over mitotic phases was not observed (Figure 5B). When analyzing the twist of metaphase spindles across the treatments, we found that in HeLa cells, a strong left-handed twist was prevalent in spindles with high width/length ratios (higher than ~0.8; Figure 5C), whereas in RPE1 cells, a strong right-handed twist was found in a subset of treatments with lower width/length ratios (lower than ~0.8, Figure 5C). Taken together, these results suggest a link between bending moments and left-handed twisting moments in HeLa cells, whereas in RPE1 cells, this relationship is less clear.

DISCUSSION

Mechanisms that generate spindle twist

In this work, we reveal biomechanical and molecular mechanisms that regulate the torques within microtubule bundles reflected in the spindle twist. From a biomechanical point of view, we show that forces within or outside the spindle regulate spindle twist (Figure 6A [box 1]). Among the spindles in HeLa cells during metaphase, round spindles are more twisted than elongated ones. In agreement with this, HeLa cell spindles in metaphase and just after anaphase onset are more round and more twisted than those in prometaphase and late anaphase, when the spindles are elongated and twist is largely absent. In RPE1 spindles, which are overall more elongated than HeLa spindles are, the twist is weaker and not correlated with the width/length ratio. Moreover, when we squeezed HeLa spindles along the pole-to-pole axis, they became rounder and their twist increased. These findings suggest that spindle roundness, which reflects bending moments within the spindle,[28] is correlated with the twist. Thus, the molecular mechanisms that generate larger bending moments, causing the spindles to be rounder, may also generate larger twisting moments, visible as stronger twists of the microtubule bundles. It is interesting to see that spindles, as complex and dynamic structures, show a relationship between twisting and bending similar to simple systems from classical beam mechanics.[59]

By performing a candidate screen, we identified several motor proteins that regulate spindle chirality, with different degrees of contribution in the following different cell lines: Eg5, Kif18A, MKLP1, and dynein. All of these motors exert torque on the microtubules *in vitro*.[34,36,37,40,42] Given that the first three motors are found within the antiparallel overlaps of bridging microtubules in the spindle,[12,54,60] we suggest that they generate the twisted shape of the bundle by rotating the antiparallel microtubules within the bundle around each other (Figure 6A [box 2]), whereas dynein acts through microtubule rotation close to the spindle pole (Figure 6A [box 3]). We propose that Eg5, which also localizes in the pole region[12,60] where it crosslinks parallel microtubules,[61,62] prevents their free rotation within the bundle, thereby promoting the accumulation of torsional stresses, though it may also actively generate torques at the pole (Figure 6A [box 3]). Additionally, other motors localized at the pole, such as Kif2a[63] and Kif2c/MCAK[64] from the kinesin-13 family, might contribute to spindle twist by rotating the microtubules with respect to the spindle pole (Figure 6A [box 3]).

Intriguingly, the strongest effect on spindle twist was obtained by depletion of the HAUS6 or HAUS8 subunits of the augmin complex, which promotes nucleation of new microtubules from the wall of existing microtubules.[48,55,65–67] Given that augmin depletion reduces the microtubule number within bridging fibers,[56] we suggest that the altered twist is related to the reduced antiparallel overlaps where torque-generating motors bind (Figure 6A [box 2]). Overexpression of the crosslinker of antiparallel microtubules PRC1 led to bundle straightening, indicating that excessive microtubule bundling due to additional PRC1 increases the torsional stiffness of the bundle (Figure 6A [box 2]). Arresting RPE1 cells in metaphase resulted in a right-handed twist, similar to the depletion of Kif18A or augmin. Interestingly,

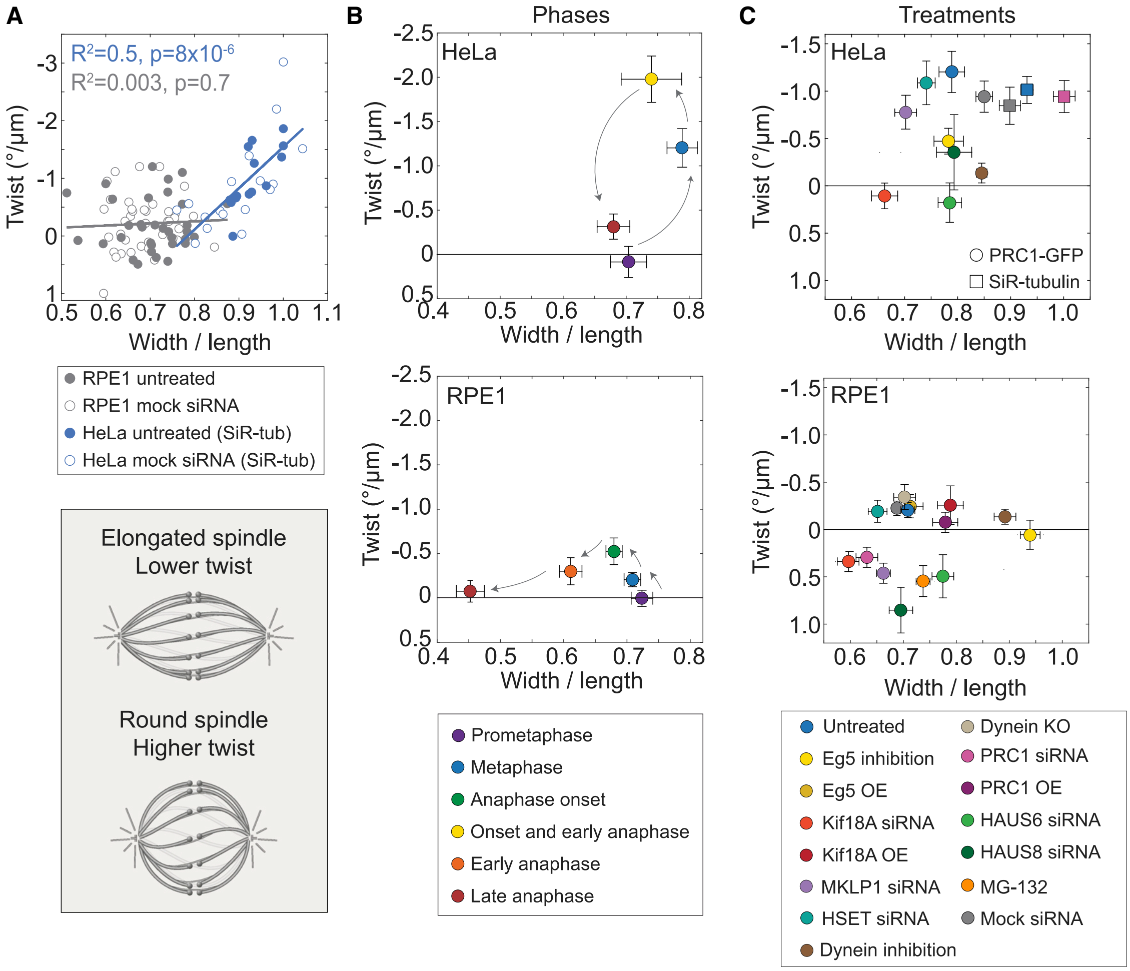

Figure 5. Round spindles have a stronger twist than elongated spindles

(A) Spindle twist versus width/length ratio in HeLa and RPE1 cells, as indicated in the legend below the graph. The lines show linear fits for each cell line (untreated cells together with mock siRNA); y = −7.16 × + 5.62 for HeLa and y = −0.36 × + 0.04 for RPE1; the goodness of fit is shown in the graph. The data for HeLa cells were also used in Figure 4B and for RPE1 cells in Figures 1E, 3C, and 4C. The cell lines used were non-transfected HeLa and hTERT-RPE1 expressing CENP-A-GFP and centrin1-GFP; for HeLa cells expressing PRC1-GFP see Figure S6A. The scheme on the bottom depicts the relationship between spindle twist and roundness.

(B) Spindle twist versus width/length ratio in HeLa-Kyoto BAC cells expressing PRC1-GFP (top) and hTERT-RPE1 cells expressing CENP-A-GFP and centrin1-GFP (bottom) over different phases of mitosis, as indicated in the legend; the error bars represent SEM; the arrows indicate progression of mitosis. The same data were used in Figures 1D and 1E. The related graphs showing twist versus spindle length and width are given in Figures S6B and S6C.

(C) Spindle twist versus width/length ratio after perturbations of spindle-associated proteins, as indicated in the legend, the error bars represent SEM. The same data were used in Figures 3B, 3C, 4B, and 4C. For HeLa cells, the experiments were performed on HeLa-Kyoto BAC cells expressing PRC1-GFP (circles) and non-transfected HeLa cells stained with SiR-tubulin (rectangles). For RPE1 cells, hTERT-RPE1 cells expressing CENP-A-GFP and centrin1-GFP were used, with the exception of RPE1 inducible CRISPR/Cas9 DYNC1H1 knockout cells in the case of "Dynein KO." The related graphs showing twist versus spindle length and width are given in Figures S6B and S6C.

these depletions also prolong metaphase,[47,48] which may contribute to the observed effect on the twist.

The observed switch of the direction of twist from left-handed to right-handed indicates the existence of competing mechanisms promoting a twist in the opposite directions. HeLa spindles have a stronger left-handed twist than RPE1 spindles have, and protein depletions that led to zero twist in HeLa cells largely resulted in a right-handed twist in RPE1 cells; thus, the twist changed in both cell lines by a similar amount. This implies that torques are regulated by similar mechanisms in both cell lines, but the torque balance is shifted more toward the formation of left-handed twist in HeLa than in RPE1 spindles. All the diverse molecular perturbations used here tuned the twist toward more positive values, suggesting that the corresponding molecular players promote a left-handed twist. We thus speculate that a right-handed twist may arise due to the activity of additional microtubule-associated proteins and/or the helical structure of the microtubule lattice.[68]

In contrast to the left-handed twist of human spindles, the spindles in the amoeba *Naegleria gruberi* are twisted in a right-handed fashion,[30] which may be due to the differences in

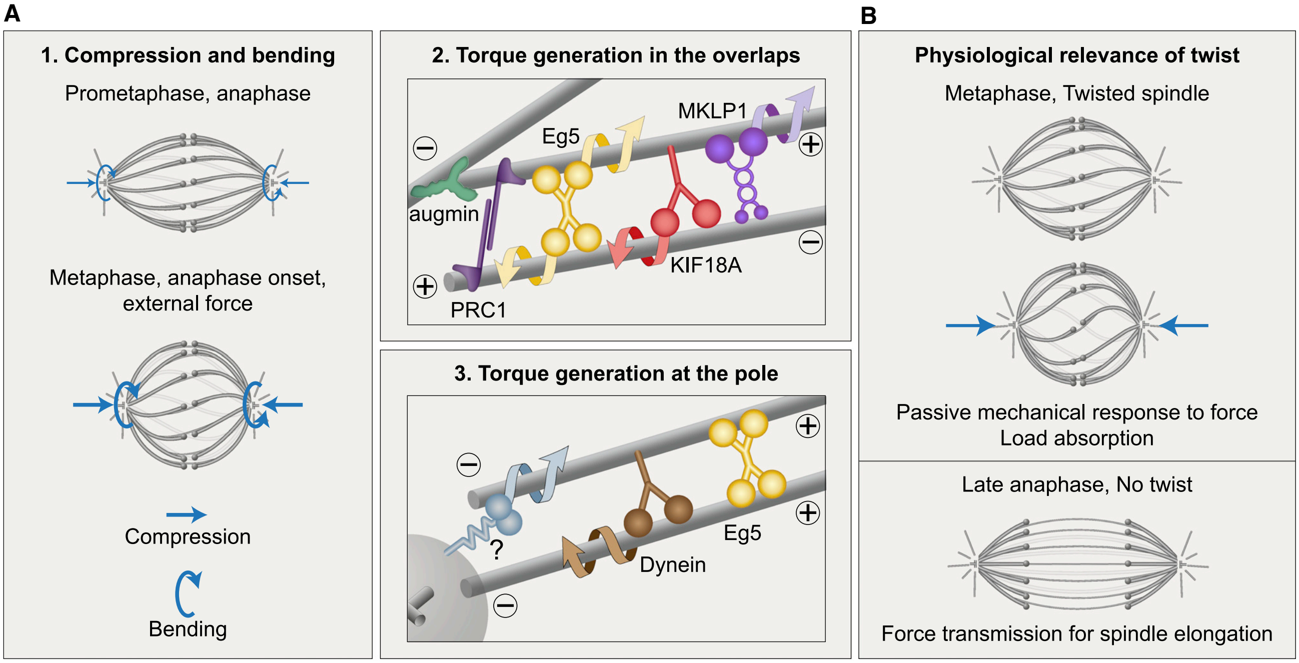

Figure 6. Biomechanical and molecular origins of spindle twist and its biological role

(A) Forces regulate twist (box 1). Round spindles or those compressed by external forces (straight arrows) are more twisted than elongated ones, suggesting that larger bending moments (curved arrows) are correlated with a larger twist. Within the antiparallel overlaps of bridging microtubules (box 2), Eg5, Kif18A, and MKLP1 rotate the microtubules around one another, whereas crosslinking by PRC1 limits free rotation of microtubules and modulates the torsional rigidity of the bundle. Augmin contributes to the twist by nucleating bridging microtubules. At the spindle pole (box 3), Eg5 crosslinks parallel microtubules, which prevents their free rotation. Eg5, dynein, and other motors (question mark) may rotate the microtubules around the pole and/or around other microtubules.

(B) Spindle twist allows for a mechanical response to external forces by absorbing load during metaphase (top). In contrast, in late anaphase, twist is absent, which promotes force transmission for spindle elongation and maintenance of chromosome separation (bottom).

spindle proteins between *Naegleria* and humans. *Naegleria* lacks homologs to subunits of the augmin complex,[69] which is in line with the right-handed twist of spindles in this amoeba and in RPE1 cells depleted of augmin. Helical structures are also widespread in plants, e.g., *lefty* mutants in *Arabidopsis thaliana* have cortical microtubule arrays that form right-handed helices, resulting in clockwise bending of leaf petioles and flower petals when viewed from above,[70] whereas *spiral* mutants show counterclockwise bending.[71] What determines the direction and amount of twist in microtubules of different organisms and whether there are common elements remain as intriguing areas for upcoming studies.

The physiological function of spindle twist

Although spindle chirality may be simply a side effect of the action of motors that generate torque, the twisted shapes of microtubule bundles may contribute to spindle function. We propose that the twisted shape observed during metaphase is beneficial for the spindle because it allows for changes of spindle shape as a mechanical response to external forces. In this picture, a twisted spindle can quickly shorten under compressive forces by increasing the twist in a manner similar to an elastic spring (Figure 6B [top]). Our experiments in which we compressed the spindle end-on and observed an increase in twist as the spindle shortened while the contour length of microtubule bundles remained largely unchanged provide support for the model in which the built-in twist helps the spindle respond to forces quickly without microtubule shortening.

In contrast to metaphase, during late anaphase, the spindle is not chiral as the bundles straighten likely due to the accumulation of PRC1 and other midzone proteins. We speculate that the straight shapes of the bundles are beneficial for the spindle in late anaphase to allow for force transmission from the central overlap region to the poles and to move the chromosomes apart and keep them separated (Figure 6B [bottom]).

Additional functions of spindle chirality may be to promote the physical separation of adjacent bundles during prometaphase or to help start spindle elongation at the onset of anaphase by releasing elastic energy stored in the twisted bundles. Intriguingly, a recent study showed that changes in twist can be associated with chromosome segregation errors.[29] Thus, the regulation of twist may also be important for the fidelity of chromosome segregation, which will be an exciting topic for further research.

STAR★METHODS

Detailed methods are provided in the online version of this paper and include the following:

- KEY RESOURCES TABLE
- RESOURCE AVAILABILITY
  - Lead contact

CellPress
OPEN ACCESS

- Materials availability
- Data and code availability
- EXPERIMENTAL MODEL AND SUBJECT DETAILS
  - Cell lines
- METHOD DETAILS
  - Sample preparation
  - Immunofluorescence
  - Spindle compression
  - Confocal microscopy
- QUANTIFICATION AND STATISTICAL ANALYSIS
  - Analysis of spindle twist
  - Analysis of spindle length and width
  - Analysis of protein expression in spindles
  - Image processing and statistical analysis

SUPPLEMENTAL INFORMATION

Supplemental information can be found online at https://doi.org/10.1016/j.cub.2022.04.035.

A video abstract is available at https://doi.org/10.1016/j.cub.2022.04.035#mmc11.

ACKNOWLEDGMENTS

We thank Ina Poser, Tony Hyman, Alexey Khodjakov, and Iain M. Cheeseman for the cell lines; Jason Stumpff and Casper C. Hoogenraad for the plasmids; Juraj Simunić, Jurica Matković, Valentina Štimac, Isabella Koprivec, Lucija Bujanić, and Iva Buljan for their contribution to the experimental work; Martina Manenica for help with the MATLAB codes; and Ivana Šarić for the drawings. We thank Nenad Pavin, Maja Novak, Josip Tambača, Matko Ljulj, and all members of the Tolić and Pavin groups for their helpful discussions. This work was funded by the European Research Council (ERC Consolidator Grant, GA Number 647077), the Croatian Science Foundation (HRZZ project IP-2019-04-5967), the Science and Innovation Grant co-financed by the European Structural and Investment Funds (ESIF) within the Operational Programme Competitiveness and Cohesion (OPCC) 2014–2020 (grant KK.01.1.1.04.0057), and the QuantiXLie Center of Excellence, a project co-financed by the Croatian Government and European Union through the European Regional Development Fund—the Competitiveness and Cohesion Operational Programme (grant KK.01.1.1.01.0004). We also acknowledge the new support from the ERC (Synergy Grant, GA Number 855158). The work of doctoral students M.T. and A.I. have been supported in part by the "Young researchers' career development project – training of doctoral students" of the Croatian Science Foundation.

AUTHOR CONTRIBUTIONS

M.T. performed experiments on RPE1 cells. B.K. and M.T. performed experiments on the HeLa cells, except for the compression experiments, which were done by I.P. M.T., B.K., and I.P. analyzed the experimental data. I.B. and S.Š. developed the optical flow method for twist measurement. A.I. measured the twist from bundle traces. M.T. and I.M.T. wrote the manuscript, with input from I.P., B.K., A.I., and other authors. M.T. assembled the figures. I.M.T. conceived the project and supervised the experiments.

DECLARATION OF INTERESTS

The authors declare no competing interests.

INCLUSION AND DIVERSITY

We worked to ensure diversity in experimental samples through the selection of the cell lines. The author list of this paper includes contributors from the location where the research was conducted who participated in the data collection, design, analysis, and/or interpretation of the work.

Received: September 23, 2021
Revised: February 17, 2022
Accepted: April 13, 2022
Published: May 9, 2022

REFERENCES

1. Nurse, P. (2000). A long twentieth century of the cell cycle and beyond. Cell *100*, 71–78.
2. McIntosh, J.R., Molodtsov, M.I., and Ataullakhanov, F.I. (2012). Biophysics of mitosis. Q. Rev. Biophys. *45*, 147–207.
3. Pavin, N., and Tolić, I.M. (2016). Self-organization and forces in the mitotic spindle. Annu. Rev. Biophys. *45*, 279–298.
4. Prosser, S.L., and Pelletier, L. (2017). Mitotic spindle assembly in animal cells: a fine balancing act. Nat. Rev. Mol. Cell Biol. *18*, 187–201.
5. Pavin, N., and Tolić, I.M. (2021). Mechanobiology of the mitotic spindle. Dev. Cell *56*, 192–201.
6. Maiato, H., Gomes, A.M., Sousa, F., and Barisic, M. (2017). Mechanisms of chromosome Congression during mitosis. Biology (Basel) *6*, 13.
7. Risteski, P., Jagrić, M., Pavin, N., and Tolić, I.M. (2021). Biomechanics of chromosome alignment at the spindle midplane. Curr. Biol. *31*, R574–R585.
8. Tanaka, T.U. (2008). Bi-orienting chromosomes: acrobatics on the mitotic spindle. Chromosoma *117*, 521–533.
9. Asbury, C.L. (2017). Anaphase A: disassembling microtubules move chromosomes toward spindle poles. Biology (Basel) *6*, 15.
10. Vukušić, K., Buđa, R., and Tolić, I.M. (2019). Force-generating mechanisms of anaphase in human cells. J. Cell Sci. *132*, jcs231985.
11. Anjur-Dietrich, M.I., Kelleher, C.P., and Needleman, D.J. (2021). Mechanical mechanisms of chromosome segregation. Cells *10*, 465.
12. Kajtez, J., Solomatina, A., Novak, M., Polak, B., Vukušić, K., Rüdiger, J., Cojoc, G., Milas, A., Šumanovac Šestak, I., Risteski, P., et al. (2016). Overlap microtubules link sister k-fibres and balance the forces on bi-oriented kinetochores. Nat. Commun. *7*, 10298.
13. Polak, B., Risteski, P., Lesjak, S., and Tolić, I.M. (2017). PRC1-labeled microtubule bundles and kinetochore pairs show one-to-one association in metaphase. EMBO Rep *18*, 217–230.
14. Vukušić, K., Buđa, R., Bosilj, A., Milas, A., Pavin, N., and Tolić, I.M. (2017). Microtubule sliding within the bridging fiber pushes kinetochore fibers apart to segregate chromosomes. Dev. Cell *43*, 11–23.e6.
15. Tolić, I.M. (2018). Mitotic spindle: kinetochore fibers hold on tight to interpolar bundles. Eur. Biophys. J. *47*, 191–203.
16. Scholey, J.M., Civelekoglu-Scholey, G., and Brust-Mascher, I. (2016). Anaphase B. Biology (Basel) *5*, 51.
17. Vukušić, K., Ponjavić, I., Buđa, R., Risteski, P., and Tolić, I.M. (2021). Microtubule-sliding modules based on kinesins EG5 and PRC1-dependent KIF4A drive human spindle elongation. Dev. Cell *56*, 1253–1267. e10.
18. Vukušić, K., and Tolić, I.M. (2021). Anaphase B: long-standing models meet new concepts. Semin. Cell Dev. Biol. *117*, 127–139.
19. Howard, J. (2001). Mechanics of Motor Proteins and the Cytoskeleton (Sunderland, Mass (Sinauer Associates).
20. Tolić-Nørrelykke, I.M. (2008). Push-me-pull-you: how microtubules organize the cell interior. Eur. Biophys. J. *37*, 1271–1278.
21. Mitchison, T., and Kirschner, M. (1984). Dynamic instability of microtubule growth. Nature *312*, 237–242.
22. Nicklas, R.B. (1983). Measurements of the force produced by the mitotic spindle in anaphase. J. Cell Biol. *97*, 542–548.
23. Rubinstein, B., Larripa, K., Sommi, P., and Mogilner, A. (2009). The elasticity of motor-microtubule bundles and shape of the mitotic spindle. Phys. Biol. *6*, 016005.
24. Schaap, I.A., Carrasco, C., de Pablo, P.J., MacKintosh, F.C., and Schmidt, C.F. (2006). Elastic response, buckling, and instability of microtubules under radial indentation. Biophys. J. *91*, 1521–1531.

25. Tolić, I.M., Novak, M., and Pavin, N. (2019). Helical twist and rotational forces in the mitotic spindle. Biomolecules *9*, 132.

26. Gittes, F., Mickey, B., Nettleton, J., and Howard, J. (1993). Flexural rigidity of microtubules and actin filaments measured from thermal fluctuations in shape. J. Cell Biol. *120*, 923–934.

27. Dogterom, M., and Yurke, B. (1997). Measurement of the force-velocity relation for growing microtubules. Science *278*, 856–860.

28. Novak, M., Polak, B., Simunić, J., Boban, Z., Kuzmić, B., Thomae, A.W., Tolić, I.M., and Pavin, N. (2018). The mitotic spindle is chiral due to torques within microtubule bundles. Nat. Commun. *9*, 3571.

29. Neahring, L., Cho, N.H., and Dumont, S. (2021). Opposing motors provide mechanical and functional robustness in the human spindle. Dev. Cell *56*, 3006–3018. e5.

30. Velle, K.B., Kennard, A.S., Trupinić, M., Ivec, A., Swafford, A.J.M., Nolton, E., Rice, L.M., Tolić, I.M., Fritz-Laylin, L.K., and Wadsworth, P. (2022). Naegleria's mitotic spindles are built from unique tubulins and highlight core spindle features. Curr. Biol. *32*, 1247–1261. e6.

31. Vale, R.D., and Toyoshima, Y.Y. (1988). Rotation and translocation of microtubules in vitro induced by dyneins from Tetrahymena cilia. Cell *52*, 459–469.

32. Walker, R.A., Salmon, E.D., and Endow, S.A. (1990). The Drosophila claret segregation protein is a minus-end directed motor molecule. Nature *347*, 780–782.

33. Yajima, J., and Cross, R.A. (2005). A torque component in the kinesin-1 power stroke. Nat. Chem. Biol. *1*, 338–341.

34. Yajima, J., Mizutani, K., and Nishizaka, T. (2008). A torque component present in mitotic kinesin Eg5 revealed by three-dimensional tracking. Nat. Struct. Mol. Biol. *15*, 1119–1121.

35. Brunnbauer, M., Dombi, R., Ho, T.H., Schliwa, M., Rief, M., and Ökten, Z. (2012). Torque generation of kinesin motors is governed by the stability of the neck domain. Mol. Cell *46*, 147–158.

36. Bormuth, V., Nitzsche, B., Ruhnow, F., Mitra, A., Storch, M., Rammner, B., Howard, J., and Diez, S. (2012). The highly processive kinesin-8, Kip3, switches microtubule protofilaments with a bias toward the left. Biophys. J. *103*. L4–L6.

37. Can, S., Dewitt, M.A., and Yildiz, A. (2014). Bidirectional helical motility of cytoplasmic dynein around microtubules. eLife *3*, e03205.

38. Bugiel, M., Böhl, E., and Schäffer, E. (2015). The Kinesin-8 Kip3 switches protofilaments in a sideward random walk asymmetrically biased by force. Biophys. J. *108*, 2019. –2027.

39. Ramaiya, A., Roy, B., Bugiel, M., and Schäffer, E. (2017). Kinesin rotates unidirectionally and generates torque while walking on microtubules. Proc. Natl. Acad. Sci. U. S. A. *114*, 10894–10899.

40. Mitra, A., Ruhnow, F., Girardo, S., and Diez, S. (2018). Directionally biased sidestepping of Kip3/kinesin-8 is regulated by ATP waiting time and motor-microtubule interaction strength. Proc. Natl. Acad. Sci. U. S. A. *115*, E7950–E7959.

41. Mitra, A., Meißner, L., Gandhimathi, R., Renger, R., Ruhnow, F., and Diez, S. (2020). Kinesin-14 motors drive a right-handed helical motion of antiparallel microtubules around each other. Nat. Commun. *11*, 2565.

42. Maruyama, Y., Sugawa, M., Yamaguchi, S., Davies, T., Osaki, T., Kobayashi, T., Yamagishi, M., Takeuchi, S., Mishima, M., and Yajima, J. (2021). CYK4 relaxes the bias in the off-axis motion by MKLP1 kinesin-6. Commun. Biol. *4*, 180.

43. Ivec, A., Trupinić, M., Tolić, I.M., and Pavin, N. (2021). Oblique circle method for measuring the curvature and twist of mitotic spindle microtubule bundles. Biophys. J. *120*, 3641–3648.

44. Liu, X., and Winey, M. (2012). The MPS1 family of protein kinases. Annu. Rev. Biochem. *81*, 561–585.

45. Hewitt, L., Tighe, A., Santaguida, S., White, A.M., Jones, C.D., Musacchio, A., Green, S., and Taylor, S.S. (2010). Sustained Mps1 activity is required in mitosis to recruit O-Mad2 to the Mad1-C-Mad2 core complex. J. Cell Biol. *190*, 25–34.

46. Dumont, S., and Mitchison, T.J. (2009). Force and length in the mitotic spindle. Curr. Biol. *19*, R749–R761.

47. Stumpff, J., von Dassow, G., Wagenbach, M., Asbury, C., and Wordeman, L. (2008). The kinesin-8 motor Kif18A suppresses kinetochore movements to control mitotic chromosome alignment. Dev. Cell *14*, 252–262.

48. Uehara, R., Nozawa, R.S., Tomioka, A., Petry, S., Vale, R.D., Obuse, C., and Goshima, G. (2009). The augmin complex plays a critical role in spindle microtubule generation for mitotic progression and cytokinesis in human cells. Proc. Natl. Acad. Sci. U. S. A. *106*, 6998–7003.

49. Zhu, C., Zhao, J., Bibikova, M., Leverson, J.D., Bossy-Wetzel, E., Fan, J.B., Abraham, R.T., and Jiang, W. (2005). Functional analysis of human microtubule-based motor proteins, the kinesins and dyneins, in mitosis/cytokinesis using RNA interference. Mol. Biol. Cell *16*, 3187–3199.

50. Skoufias, D.A., DeBonis, S., Saoudi, Y., Lebeau, L., Crevel, I., Cross, R., Wade, R.H., Hackney, D., and Kozielski, F. (2006). S-trityl-L-cysteine is a reversible, tight binding inhibitor of the human kinesin Eg5 that specifically blocks mitotic progression. J. Biol. Chem. *281*, 17559–17569.

51. Höing, S., Yeh, T.Y., Baumann, M., Martinez, N.E., Habenberger, P., Kremer, L., Drexler, H.C.A., Küchler, P., Reinhardt, P., Choidas, A., et al. (2018). Dynarrestin, a novel inhibitor of cytoplasmic dynein. Cell Chem. Biol. *25*, 357–369. e6.

52. McKinley, K.L., and Cheeseman, I.M. (2017). Large-scale analysis of CRISPR/Cas9 cell-cycle knockouts reveals the diversity of p53-dependent responses to cell-cycle defects. Dev. Cell *40*, 405–420.e2.

53. Jiang, W., Jimenez, G., Wells, N.J., Hope, T.J., Wahl, G.M., Hunter, T., and Fukunaga, R. (1998). PRC1: a human mitotic spindle-associated CDK substrate protein required for cytokinesis. Mol. Cell *2*, 877–885.

54. Jagrić, M., Risteski, P., Martinčić, J., Milas, A., and Tolić, I.M. (2021). Optogenetic control of PRC1 reveals its role in chromosome alignment on the spindle by overlap length-dependent forces. eLife *10*, e61170.

55. David, A.F., Roudot, P., Legant, W.R., Betzig, E., Danuser, G., and Gerlich, D.W. (2019). Augmin accumulation on long-lived microtubules drives amplification and kinetochore-directed growth. J. Cell Biol. *218*, 2150–2168.

56. Manenica, M., Štimac, V., Koprivec, I., Simunic, J., and Tolic, I.M. (2020). Augmin Regulates Kinetochore Tension and Spatial Arrangement of Spindle Microtubules by Nucleating Bridging Fibers. bioRxiv, https://doi.org/10.1101/2020.09.10.291740.

57. Wu, G., Lin, Y.T., Wei, R., Chen, Y., Shan, Z., and Lee, W.H. (2008). Hice1, a novel microtubule-associated protein required for maintenance of spindle integrity and chromosomal stability in human cells. Mol. Cell. Biol. *28*, 3652–3662.

58. Song, J.G., King, M.R., Zhang, R., Kadzik, R.S., Thawani, A., and Petry, S. (2018). Mechanism of how augmin directly targets the gamma-tubulin ring complex to microtubules. J. Cell Biol. *217*, 2417–2428.

59. Landau, L.D., Lifshitz, E.M., Kosevich, A.M., Sykes, J.B., Pitaevskii, L.P., and Reid, W.H. (1986). Theory of Elasticity, *7* (Elsevier Science).

60. Mann, B.J., and Wadsworth, P. (2018). Distribution of Eg5 and TPX2 in mitosis: insight from CRISPR tagged cells. Cytoskeleton (Hoboken) *75*, 508–521.

61. Valentine, M.T., Fordyce, P.M., and Block, S.M. (2006). Eg5 steps it up. Cell Div *1*, 31.

62. Walczak, C.E., Vernos, I., Mitchison, T.J., Karsenti, E., and Heald, R. (1998). A model for the proposed roles of different microtubule-based motor proteins in establishing spindle bipolarity. Curr. Biol. *8*, 903–913.

63. Jang, C.Y., Wong, J., Coppinger, J.A., Seki, A., Yates, J.R., 3rd, and Fang, G. (2008). DDA3 recruits microtubule depolymerase Kif2a to spindle poles and controls spindle dynamics and mitotic chromosome movement. J. Cell Biol. *181*, 255–267.

64. Ganguly, A., Bhattacharya, R., and Cabral, F. (2008). Cell cycle dependent degradation of MCAK: Evidence against a role in anaphase chromosome movement. Cell Cycle *7*, 3187–3193.

CellPress
OPEN ACCESS

65. Goshima, G., Mayer, M., Zhang, N., Stuurman, N., and Vale, R.D. (2008). Augmin: a protein complex required for centrosome-independent microtubule generation within the spindle. J. Cell Biol. *181*, 421–429.
66. Kamasaki, T., O'Toole, E., Kita, S., Osumi, M., Usukura, J., McIntosh, J.R., and Goshima, G. (2013). Augmin-dependent microtubule nucleation at microtubule walls in the spindle. J. Cell Biol. *202*, 25–33.
67. Petry, S., Groen, A.C., Ishihara, K., Mitchison, T.J., and Vale, R.D. (2013). Branching microtubule nucleation in Xenopus egg extracts mediated by augmin and TPX2. Cell *152*, 768–777.
68. Amos, L.A., and Hirose, K. (2007). Studying the structure of microtubules by electron microscopy. Methods Mol. Med. *137*, 65–91.
69. Fritz-Laylin, L.K., Prochnik, S.E., Ginger, M.L., Dacks, J.B., Carpenter, M.L., Field, M.C., Kuo, A., Paredez, A., Chapman, J., Pham, J., et al. (2010). The genome of Naegleria gruberi illuminates early eukaryotic versatility. Cell *140*, 631–642.
70. Thitamadee, S., Tuchihara, K., and Hashimoto, T. (2002). Microtubule basis for left-handed helical growth in Arabidopsis. Nature *417*, 193–196.
71. Furutani, I., Watanabe, Y., Prieto, R., Masukawa, M., Suzuki, K., Naoi, K., Thitamadee, S., Shikanai, T., and Hashimoto, T. (2000). The SPIRAL genes are required for directional control of cell elongation in Aarabidopsis thaliana. Development *127*, 4443–4453.
72. Schindelin, J., Arganda-Carreras, I., Frise, E., Kaynig, V., Longair, M., Pietzsch, T., Preibisch, S., Rueden, C., Saalfeld, S., Schmid, B., et al. (2012). Fiji: an open-source platform for biological-image analysis. Nat. Methods *9*, 676–682.
73. Poser, I., Sarov, M., Hutchins, J.R., Hériché, J.K., Toyoda, Y., Pozniakovsky, A., Weigl, D., Nitzsche, A., Hegemann, B., Bird, A.W., et al. (2008). BAC TransgeneOmics: a high-throughput method for exploration of protein function in mammals. Nat. Methods *5*, 409–415.
74. Magidson, V., O'Connell, C.B., Lončarek, J., Paul, R., Mogilner, A., and Khodjakov, A. (2011). The spatial arrangement of chromosomes during prometaphase facilitates spindle assembly. Cell *146*, 555–567.
75. Buđa, R., Vukušić, K., and Tolić, I.M. (2017). Dissection and characterization of microtubule bundles in the mitotic spindle using femtosecond laser ablation. Methods Cell Biol *139*, 81–101.
76. Lowe, D.G. (2004). Distinctive image features from scale-invariant keypoints. Int. J. Comput. Vision *60*, 91–110.
77. Jin, F., and Feng, D.Z. (2014). Image registration algorithm using Mexican hat function-based operator and grouped feature matching strategy. PLOS ONE *9*, e95576.
78. Farnebäck, G. (2003). Two-frame motion estimation based on polynomial expansion. Lecture Notes in Computer Science (Springer)), pp. 363–370.

STAR★METHODS

KEY RESOURCES TABLE

REAGENT or RESOURCE	SOURCE	IDENTIFIER
**Antibodies**		
Mouse monoclonal anti-PRC1 antibody (C-1)	Santa Cruz Biotechnology	Cat# sc-376983;
Rabbit polyclonal anti-KIF18A antibody	Bethyl Laboratories	Cat# A301-080A; RRID: AB_2296551
Rabbit polyclonal anti-FAM28A antibody	Abcam	Cat# ab150806;
Rabbit polyclonal anti-HICE1 antibody	Thermo Fisher	Cat# PA5-21331; RRID: AB_11153508
Mouse monoclonal anti-KIFC1 antibody (M-63)	Santa Cruz Biotechnology	Cat# sc-100947; RRID: AB_2132540
Rabbit monoclonal anti-MKLP1 antibody (EPR10879)	Abcam	Cat# ab174304;
AF594 donkey anti-mouse preadsorbed secondary antibody	Abcam	Cat# ab150112; RRID: AB_2813898
AF594 donkey anti-rabbit preadsorbed secondary antibody	Abcam	Cat# ab150064; RRID: AB_2734146
**Chemicals, peptides, and recombinant proteins**		
Dulbecco's modified Eagle's medium (DMEM)	Capricorn Scientific GmbH	Cat# DMEM-HPSTA
Fetal bovine serum (FBS), sterile-filtered	Sigma Aldrich	Cat# F2442
Penicillin/streptomycin solution (Pen/Strep)	Capricorn Scientific GmbH	Cat# PS-B
Geneticin selective antibiotic (G418 Sulfate)	Thermo Fisher	Cat# 10131027
Doxycycline hyclate	Sigma-Aldrich	Cat# D9891-1G
Silicone rhodamine (SiR)-tubulin	Spirochrome AG	Cat# SC002
Silicone rhodamine (SiR)-DNA	Spirochrome AG	Cat# SC007
NucBlue Live Ready Probes Reagent (Hoechst 33342)	Thermo Fisher	Cat# R37605
(+)-S-Trityl-L-cysteine (STLC)	Sigma-Aldrich	Cat# 164739-5G
Dynarrestin inhibitor (HY-121802)	MedChemExpress	Cat# 50-202-9915
Mps1 inhibitor AZ3146	Sigma Aldrich	Cat# SML1427-5MG
MG-132	Sigma Aldrich	Cat# M7449-1ML
Paraformaldehyde 4%	Santa Cruz Biotechnology	Cat# sc-281692
Glutaraldehyde 50%	Sigma Aldrich	Cat# G7651
$MgCl_2$	Sigma Aldrich	Cat# M8266
PIPES	Sigma Aldrich	Cat# P6757-500G
EDTA	Sigma Aldrich	Cat# EDS
Triton-X-100	Sigma Aldrich	Cat# 93426
Immunopure Normal Goat Serum (iNGS)	Thermo Fisher	Cat# 31872
Phosphate-buffered saline	Dulbecco	Cat# L 182 50
Borohydride	Sigma Aldrich	Cat# 213462
Methanol 100%	Sigma Aldrich	Cat# 32213-2.5L-M
Ultra-pure agarose	Thermo Fisher	Cat# 15510
Trypsin/EDTA	Biochrom AG	N/A
**Critical commercial assays**		
MycoAlert mycoplasma detection kit	Lonza	Cat# LT07-218
Lipofectamine RNAiMAX Reagent	Thermo Fisher	Cat# 13778150
Amaxa Cell Line Nucleofactor Kit R	Lonza	Cat# VCA-1001

(*Continued on next page*)

Continued

REAGENT or RESOURCE	SOURCE	IDENTIFIER
Experimental models: Cell lines		
human HeLa cell line (human adenocarcinoma, female) permanently transfected and stabilized using BAC containing PRC1-GFP	Max Planck Institute of Molecular Cell Biology and Genetics, Dresden, Germany	N/A
human unlabeled (non-transfected, female) HeLa-TDS cells from the High-Throughput Technology Development Studio	MPI-CBG, Dresden	N/A
human hTERT-RPE1 (retinal pigmented epithelium, female) permanently transfected and stabilized using CENP-A-GFP and centrin1-GFP (protein of a centrosome complex)	Wadsworth Center, New York State Department of Health, Albany, NY	N/A
human hTERT-RPE1 inducible CRISPR/Cas9/ DYNC1H1 knock-out (KO)	Massachusetts Institute of Technology, Cambridge, MA, USA	N/A
Oligonucleotides		
human Silencer Select Validated Kif18A siRNA	Thermo Fisher	Cat# 4390824
human ON-TARGETplus SMARTpool PRC1 siRNA	Dharmacon	Cat# L-C19491-00-0010
human ON-TARGETplus SMARTpool HAUS6 siRNA	Dharmacon	Cat# L-018372-01-0005
human ON-TARGETplus SMARTpool HAUS8 siRNA	Dharmacon	Cat# L-031247-01-0005
human ON-TARGETplus SMARTpool KIFC1 siRNA	Dharmacon	Cat# L-004958-00
human MKLP1 siRNA	Santa Cruz Biotechnology	Cat# sc-35936
human ON-TARGETplus Control Pool Non-Targeting pool siRNA	Dharmacon	Cat# D-001810-10-05
human Silencer Select Negative Control #1 siRNA	Thermo Fisher	Cat# 4390843
Recombinant DNA		
mEmerald-Kinesin11-N-18 plasmid	Addgene	#54137
EGFP-Kif18A plasmid	Laboratory of Jason Stumpff, University of Vermont, Burlington, VT, USA	N/A
PRC1-mCherry plasmid	Laboratory of Casper C. Hoogenraad, Utrecht University, Utrecht, Netherland	N/A
Software and algorithms		
ImageJ	[72]	https://imagej.nih.gov/ij/
Matlab	The Mathworks, Inc.	https://nl.mathworks.com/products/matlab.html
Adobe Illustrator CS6 and Adobe Photoshop CS6	Adobe Systems, Mountain View, CA, USA	https://www.adobe.com/
Python	Python Software Foundation	https://www.python.org/
R Studio	RStudio, PBC	https://www.rstudio.com/
Other		
Optical flow code for twist analysis	This paper	GitLab: https://gitlab.com/IBarisic/detecting-microtubules-helicity-in-microscopic-3d-images

RESOURCE AVAILABILITY

Lead contact

Further information and requests for resources should be directed to and will be fulfilled by the lead contact, Iva M. Tolić (tolic@irb.hr).

Materials availability

This study did not generate new unique reagents.

Data and code availability

The datasets generated in this study will be made available on request from the lead contact without restrictions. All original code has been deposited at GitLab and is publicly available as of the date of publication. DOIs are listed in the key resources table. Any additional information required to reanalyze the data reported in this paper is available from the lead contact upon request.

EXPERIMENTAL MODEL AND SUBJECT DETAILS

Cell lines

The human cell lines used are: **1**. human HeLa cell line (human adenocarcinoma, female) permanently transfected and stabilized using BAC containing PRC1-GFP which was a gift from Ina Poser and Tony Hyman (Max Planck Institute of Molecular Cell Biology and Genetics, Dresden, Germany),[73] **2**. human unlabeled (non-transfected, female) HeLa-TDS cells from the High-Throughput Technology Development Studio (MPI-CBG, Dresden), **3**. human hTERT-RPE1 (retinal pigmented epithelium, female) permanently transfected and stabilized using CENP-A-GFP and centrin1-GFP (protein of a centrosome complex), which was a gift from Alexey Khodjakov (Wadsworth Center, New York State Department of Health, Albany, NY),[74] **4**. human hTERT-RPE1 inducible CRISPR/Cas9/DYNC1H1 knock-out (KO) which was a gift from Iain Cheeseman (Massachusetts Institute of Technology, Cambridge, MA, USA).[52] Cells were grown in flasks in Dulbecco's modified Eagle's medium (DMEM; Capricorn Scientific GmbH, Germany) supplemented with 10% fetal bovine serum (FBS; Sigma-Aldrich, MO, USA), 10000 U/ml penicillin/streptomycin solution (Capricorn Scientific GmbH, Germany), and for HeLa-Kyoto BAC cells also 50 μg/mL geneticin (Life Technologies, Waltham, MA, USA). CRISPR/Cas9 knockout of DYNC1H1 in RPE1 cell line was induced with doxycycline hyclate (D9891-1G, Sigma-Aldrich, MO, USA) at the final concentration of 1 μg/mL at 24 hour intervals for 4 consecutive days, with imaging and analysis on the fifth day. The cells were kept at 37 °C and 5% CO2 in a Galaxy 170S CO2 humidified incubator (Eppendorf, Hamburg, Germany) and regularly passaged at the confluence of 70%–80%. All used cell lines were confirmed to be mycoplasma free by monthly checks using MycoAlert Mycoplasma Detection Kit (Lonza) and regular checks during imaging experiments with DNA labelling stains.

METHOD DETAILS

Sample preparation

To visualize microtubules in experiments on RPE1 and non-transfected HeLa cells, silicon rhodamine (SiR)-tubulin (λ_{Abs} 652 nm, λ_{Em} 674 nm) (Spirochrome AG, Stein am Rhein, Switzerland) dye was added to the dish at the final concentration of 100 nM, 2-3 hours prior to imaging. To visualize chromosomes and determine phase of the mitosis of the spindle in experiments on HeLa PRC1-GFP cells, 1 hour prior to imaging silicon rhodamine (SiR)-DNA (λ_{Abs} 652 nm, λ_{Em} 674 nm) (Spirochrome AG, Stein am Rhein, Switzerland) was added to the dish at a final concentration of 100 nM. To visualize chromosomes and determine phase of the mitosis of the spindles in experiments on non-transfected HeLa cells and RPE1 inducible DYNC1H1 knockout cells, 50 μL of NucBlue Live Ready Probes Reagent (Hoechst 33342) (Invitrogen by Thermo Fischer Scientific, MA, USA) dye was added to the dishes, 1 min before imaging.

Lipofectamine RNAiMAX reagent (Invitrogen by Thermo Fisher Scientific, MA, USA) was used for RNAi treatments following manufacturer's instructions. Transfections with siRNA were always performed 48 hours prior to imaging at the final concentration of 100 nM. For the inhibition of Eg5, cells were treated with (+)-S-Trityl-L-cysteine (STLC, Sigma-Aldrich, MO, USA) at the final concentration of 40 μM right before the imaging so that cells are not yet collapsed into a monopol during imaging. STLC-treated cells were imaged before spindle shortening (up to 5 min in STLC) and after shortening (10-20 min in STLC). For the inhibition of dynein, cells were treated with dynarrestin (HY-121802/CS-0083323, MedChemExpress, NJ, USA) at the final concentration of 50 μM, 1 hour prior to imaging, and were imaged up to 2 hours after the addition of the drug. This time period allowed the spindles to shorten, which was used to confirm that the inhibition experiment worked.[51] For depletion of endogenous Kif18A, cells were transfected with Kif18A Silencer Select siRNA (4390824, Ambion, Thermo Fisher Scientific, MA, USA). For depletion of endogenous PRC1, cells were transfected with ON-TARGETplus SMARTpool Human PRC1 (L-C19491-00-0010, Dharmacon, CO, USA). For depletions of endogenous HAUS6 and HAUS8, cells were transfected with ON-TARGETplus SMARTpool Human HAUS6 (L-018372-01-0005, Dharmacon, CO, USA) and ON-TARGETplus SMARTpool Human HAUS8 (L-031247-01-0005, Dharmacon, CO, USA), respectively. For the depletion of endogenous HSET, cells were transfected with ON-TARGETplus SMART pool Human KIFC1 (L-004958-00, Dharmacon, CO, USA). For the depletion of endogenous MKLP1, cells were transfected with siRNA (sc-35936; Santa Cruz Biotechnology, TX, USA). In mock experiments cells were transfected with equal amount of ON-TARGETplus Control Pool Non-Targeting pool (D-001810-10-05, Dharmacon, CO, USA) or Silencer Select Negative Control #1 siRNA (4390843, Ambion, Thermo Fisher Scientific, MA, USA).

All plasmid transfections were performed using Nucleofactor Kit R with the Nucleofactor 2b Device (Lonza, Basel, Switzerland) using Y-001 program for human HMEC cells (high efficiency). To overexpress Eg5 protein, cells were transfected with 5 μg of mEmerald-Kinesin11-N-18 plasmid (Addgene number: 54137) 24 hours prior to imaging. For Kif18A overexpression, cells were transfected with 5 μm of EGFP-Kif18A plasmid that was a gift from Jason Stumpff (University of Vermont, Burlington, VT, USA). To overexpress PRC1 protein, cells were transfected with 5 μg of mCherry-PRC1 plasmid that was a gift from Casper C. Hoogenraad (Utrecht University, Utrecht, Netherlands).

To reduce the time in which HeLa cells reach anaphase, the Mps1 inhibitor AZ3146 (Sigma-Aldrich, MO, USA) was added in prometaphase at a final concentration of 4 μM. Metaphase arrest in RPE1 cells was performed with the proteasome inhibitor MG-132 (M7449, Sigma-Aldrich, MO, USA) added at least 2 hours prior to imaging at a final concentration of 20 μM.

All experiments were performed at least three times in both cell lines, except Kif18A overexpression that was preformed once. To prepare samples for microscopy, RPE1 and HeLa cells were seeded and cultured in DMEM medium with supplements at 37 °C and 5% CO2 on uncoated 35-mm glass coverslip dishes with 0.17-mm (1.5 coverglass) glass thickness (MatTek Corporation, Ashland, MA, USA).

Immunofluorescence

HeLa-Kyoto BAC cell line stably expressing PRC1-GFP were grown on glass-bottomed dishes (14 mm, No. 1.5, MatTek Corporation) and fixed by a microtubule-preserving mixture of 3.2% PFA (paraformaldehyde) and 0.25% GA (glutaraldehyde) in microtubule-stabilizing PEM buffer (0.1 M PIPES, 0.001 M $MgCl_2$ x 6 H_2O, 0.001 M EDTA, 0.5% Triton-X-100) for 10 min at room temperature. After fixation with PFA and GA, for quenching, cells were incubated in 1 mL of freshly prepared 0.1% borohydride in PBS (phosphate-buffered saline) for 7 min and after that in 1 mL of 100 mM NH_4Cl and 100 mM glycine in PBS for 10 min at room temperature. Cells were then washed with 1 mL of PBS, 3 times for 5 min. To block unspecific binding of antibodies, cells were incubated in 500 μL blocking/permeabilization buffer (2% normal goat serum (NGS) and 0.5% Triton-X-100 in water) for 45 min at room temperature. Cells were then incubated in 500 μL of primary antibody solution (rat anti-alpha Tubulin YL1/2 (MA1-80017, Invitrogen, CA, SAD), diluted 1:500) for 24 hours at 4 °C. After primary antibody, cells were washed in PBS and then incubated in 500 μL of secondary antibody solution (donkey anti-rat IgG Alexa Fluor 594 (ab150156, Abcam), diluted 1:1000) for 45 min at room temperature.

Human hTERT-RPE1 cells, permanently transfected and stabilized using CENP-A-GFP and centrin1-GFP, were grown on glass-bottomed dishes (as described above) and fixed in cold 100% methanol for 1 min on the ice block. After fixation, cells were washed in PBS 3 times for 5 min at room temperature. Next, cells were additionally permeabilized in 0.5% Triton-X-100 solution for 15 min at room temperature and then washed in PBS (as described above). To block unspecific binding of antibodies, cells were incubated in 1% NGS solution for 1 hour on 4 °C. After washing in PBS once, cells were incubated with primary antibodies (dilution 1:100 in 1% NGS) overnight on 4 °C. Next, cells were washed in PBS 3 times for 5 min at room temperature and incubated with secondary antibodies (dilution 1:250 in 2% NGS solution) for 1 hour at room temperature covered with aluminum foil. Before microscopy, cells were washed in PBS (as described above) and left in PBS during imaging. Cells were kept in the dark in PBS on 4 °C. Primary antibodies used: PRC1 (C-1) mouse monoclonal IgG_1 (sc-376983, Santa Cruz Biotechnology, TX, USA), Rabbit anti-KIF18A Affinity Purified (A301-080A, Bethyl, TX, USA), Rb pAb to FAM29A (ab150806, Abcam, Cambridge, UK), HICE1 Polyclonal Antibody (PA5-21331, Invitrogen, MA, USA), KIFC1 (M-63) mouse monoclonal IgG_{2a} (sc-100947, Santa Cruz Biotechnology, TX, USA), Rb pAb to MKLP1 (ab174304, Abcam, Cambridge, UK); secondary antibodies used: Dnk pAb to Ms IgG (ab150112, Abcam, Cambridge, UK), Dnk pAb to Rb IgG (ab150064, Abcam, Cambridge, UK).

Spindle compression

Spindle compression method was optimized from Mitchison and Dumont, 2009.[46] A solution of 2% ultra-pure agarose (15510 Invitrogen by Thermo Fisher Scientific, MA, USA) in PBS was prepared, brought to boil and 2 mL was put in a 35 mm petri dish to solidify with ~2 mm thickness. A 1 cm × 1 cm pad area was cut out, soaked in L-15 medium overnight at 4 °C for equilibration, and warmed to 37 °C just before use. Cells were plated on 14 or 20 mm glass microwell uncoated dishes before imaging. A flat metaphase cell was chosen among 80-100% confluent cells for pre-perturbation imaging. After imaging of the metaphase cell before compression, the pad was deposited gently, centered on the cell. Note: it is important to do this step gently and with minimal moving of the dish so the position of the cell could stay intact. Using an oil hydraulic fine manipulator (InjectMan 4, micromanipulator with dynamic movement control, 100–240 V/50–60 Hz) and a coarse manipulator attached to the confocal microscope. A metal rod (which is a part of micromanipulator where the needle for microinjection is inserted) was centered on the cell and lowered (z-axis) until weak contact was made with the pad (rod diameter ≫ cell diameter). The rod was lowered slowly (over ~10 s) for several μm until the cell area expanded, and its position kept constant as the cell and spindle responses were imaged. HeLa PRC1-GFP cells were imaged every 30 s for 3 times which gave us 4 different times the cell was imaged at: before compression, 0.5 min after compression, 1 min after compression and 1.5 min after compression. Cell health was monitored through the presence of the intact cell membrane and the ability of the cell to enter anaphase after perturbation. Rough estimate of the number of vertically oriented metaphase spindles in a 35 mm petri dish is around 1 spindle per 50-100 horizontally oriented metaphase spindles. From 31 spindles that were compressed in about 45 independent experiments, 23 spindles rotated during the first 30 s of compression and could not be used for further analysis. From 8 spindles that remained vertically oriented during compression, 2 spindles were not compressed enough which was determined by their length. Successful compression, where spindle length decreased for 0.5 μm or more, was achieved on 6 spindles from 5 independent experiments, which were then used for the analysis.

Confocal microscopy

Live RPE1 and HeLa cells were imaged using Bruker Opterra Multipoint Scanning Confocal Microscope[75] (Bruker Nano Surfaces, Middleton, WI, USA). The system was mounted on a Nikon Ti-E inverted microscope equipped with a Nikon CFI Plan Apo VC ×100/1.4 numerical aperture oil objective (Nikon, Tokyo, Japan). During imaging, cells were maintained at 37 °C in Okolab Cage Incubator (Okolab, Pozzuoli, NA, Italy). A 22 μm slit aperture was used for RPE1 and 60 μm pinhole for HeLa cells. The xy-pixel

size was 83 nm. For excitation of GFP and mCherry fluorescence, a 488 and a 561 nm diode laser line was used, respectively. For SiR-dyes, a 640 nm diode laser line was used. The excitation light was separated from the emitted fluorescence by using Opterra Dichroic and Barrier Filter Set 405/488/561/640. Images were captured with an Evolve 512 Delta EMCCD Camera (Photometrics, Tucson, AZ, USA) with no binning performed. To cover the whole metaphase spindle, z-stacks were acquired at 30–60 focal planes separated by 0.5 μm with unidirectional xyz scan mode. The system was controlled with the Prairie View Imaging Software (Bruker Nano Surfaces, Middleton, WI, USA).

QUANTIFICATION AND STATISTICAL ANALYSIS

Analysis of spindle twist

To calculate spindle twist, microscopy images of horizontal spindles were analyzed in Fiji Software (ImageJ, National Institutes of Health, Bethesda, MD, USA).[72] Only images with both spindle poles in the same plane before and during imaging of the z-stack were used in analysis to avoid unspecific spindle movements in the calculation of spindle twist. Horizontal spindles were transformed into vertical orientation using a code written in R programming language in RStudio.[28] In transformed stack microtubule bundles and poles appear as blobs.

Visual assessment

In this method, the spindle is observed end-on and the rotation of microtubule bundles around the pole-to-pole axis is estimated visually. If the bundles rotate clockwise when moving along the spindle axis in the direction towards the observer, the twist is left-handed, and vice versa (Figure S1A, left). The outcome of our visual assessment is a score of spindle twist, which describes whether the spindle has a left-handed, weak left-handed, right-handed, weak right-handed, or no visible twist. Weak left-handed or weak right-handed twists correspond to a range of approximately -1 to -2 °/μm in the bundle tracing method. This is visible as a total rotation of 5-10° in the clockwise (left-handed) or counter-clockwise (right-handed) direction in the end-on view of the spindle when moving towards the observer along the bundle length, where bundles are typically 5 μm long. Left-handed or right-handed twists correspond to a rotation of more than 10° in the end-on view. The advantage of this method is its trustworthiness because coarse classification of spindles into 5 groups is reliable, whereas the main disadvantage is that the results are semi-quantitative rather than quantitative.

Optical flow

In the optical flow method, the movement of the signal coming from microtubule bundles is estimated automatically by comparing the signal from one z-plane to the next (Figure S1A, middle). This method yields a value for the average twist of all bundles in a spindle. It is the preferred choice for experiments on a large number of spindles because it is automated. Disadvantages are that it provides only the average twist value rather than the twist of each bundle, and that the results are sensitive to unspecific signal in the images, individual bundles with atypical behavior, and imperfect alignment of the spindle axis with the z-axis.

First, parts of the images containing the blobs were selected for analysis using Rectangle tool in ImageJ. In all transformed stacks only images between spindle poles were used for analysis. Transformed spindle images contained a lot of noise that was removed by using the Mexican hat filter and a threshold. The Mexican hat filter, also called the LoG (Laplacian of Gaussian) filter, was used for detection of blobs.[76,77] After applying the Mexican hat filter, a threshold was applied to the image. It removes all the pixels with intensity lower than the given threshold. Microtubule bundles of transformed spindles were detected and traced automatically using optical flow for calculating the movement of pixels between two consecutive images. Farnebäck's two-frame motion estimation algorithm (dense optical flow algorithm) was used.[78] The spindle poles were tracked manually using Multipoint tool in ImageJ. In CRISPR/Cas9 DYNC1H1 knockout RPE1 cell line, only spindles with splayed poles but bipolar shape were imaged, and the pole positions were determined visually as the outermost points of the spindle along the central spindle axis, which was defined as a line perpendicular to the metaphase plate passing through its center. Helicities of spindles were calculated using the algorithm called "All pixels weighted helicity algorithm". It calculates the total helicity as the average helicity of all pixels in the spindle, weighted by their normalized intensity. The tilt of the spindle with regard to the imaging plane was calculated from the tracked spindle poles, and the twist measurement was corrected by this tilt angle. The code for tracing of bundles and helicity calculating was written in Python programming languange using PyCharm IDE. The external libraries used in image preprocessing, calculating helicity and visualisation are NumPy, scikit-image, Matplotib, PIL, OpenCV and SciPy. The code and instructions are available at GitLab: https://gitlab.com/IBarisic/detecting-microtubules-helicity-in-microscopic-3d-images.

Bundle tracing

Bundles in images of spindles oriented vertically were traced manually using Multipoint tool in Fiji.[28] We convert the imaging plane (z-plane) to its corresponding z-coordinate by multiplying with the distance between successive planes set during image acquisition (0.5 μm) and by a factor of 0.81 to correct for the refractive index mismatch.[28] Next, to describe the shape of a microtubule bundle, we use the Oblique circle method.[43] We first position the spindle so that the pole-to-pole axis is aligned with our coordinate system, i.e., we untilt the spindle. Next, we fit a plane to the points representing the bundle, and then we fit a circle that lies in this plane to the same points. From these fits we calculate the curvature and twist of the bundle as follows: (i) The curvature is calculated as one over the radius, and (ii) the twist is calculated as the angle between the plane and the z-axis divided by the mean distance of these points from the z-axis. Contour length of the bundle was calculated as the length of the fitted circular arc plus the distance of bundle ends from the corresponding poles. The main advantage of this method is that it yields a value of twist for each individual bundle in the spindle, whereas the main disadvantage is that it requires manual tracing, which makes it impractical for high-throughput studies.

CellPress
OPEN ACCESS

Analysis of spindle length and width

To measure spindle length and width, we used the Line tool in Fiji Software (ImageJ, National Institutes of Health, Bethesda, MD, USA).[72] Length was measured by drawing a line from pole to pole of the spindle. Width in HeLa cells expressing PRC1-GFP was measured by drawing a line across the equatorial plane of the spindle, with the line ending at the outer edges of a spindle. Width in RPE1 cells expressing CENP-A-GFP and centrin1-GFP was measured by drawing a line across the equatorial plane of the spindle, with the line ending at the outer kinetochore pairs.

Analysis of protein expression in spindles

To quantify protein expression, the fluorescence intensity signal of the protein of interest was measured on the whole spindle region using ImageJ Polygon Selection tool Software (ImageJ, National Institutes of Health, Bethesda, MD, USA)[72] on the sum-intensity projection of the whole z-stack. The mean background fluorescence intensity measured in the cytoplasm was subtracted from the mean value obtained on the spindle, and the resulting value was divided by the number of z-slices used in the sum projection.

Image processing and statistical analysis

Fiji was used to scale images and adjust brightness and contrast. Figures were assembled in Adobe Illustrator CS5 and CC (Adobe Systems, Mountain View, CA, USA). Graphs were plotted in MATLAB (MathWorks, Natick, MA, USA). For generation of univariate scatter plots, the open "UnivarScatter" Matlab extension was used (https://github.com/manulera/UnivarScatter). Data are given as mean ± SEM, unless otherwise stated. Significance of data was estimated by Student's t-test (two-tailed and two sample unequal-variance; except for the experiments with spindle compression, where a paired t-test was used to compare the values for the same spindles before and after compression). $p < 0.05$ was considered statistically significant. Values of all significant differences are given with degree of significance indicated (*$0.01 < p < 0.05$, **$0.001 < p < 0.01$, ***$p < 0.001$). Statistically significant differences between groups of data were determined by one-way ANOVA and Tukey's HSD post hoc test, $p < 0.05$ was considered statistically significant. The number of analyzed cells and microtubule bundles is given in the respective figure panel.

Current Biology, Volume *32*

Supplemental Information

The chirality of the mitotic spindle provides a mechanical response to forces and depends on microtubule motors and augmin

Monika Trupinić, Barbara Kokanović, Ivana Ponjavić, Ivan Barišić, Siniša Šegvić, Arian Ivec, and Iva M. Tolić

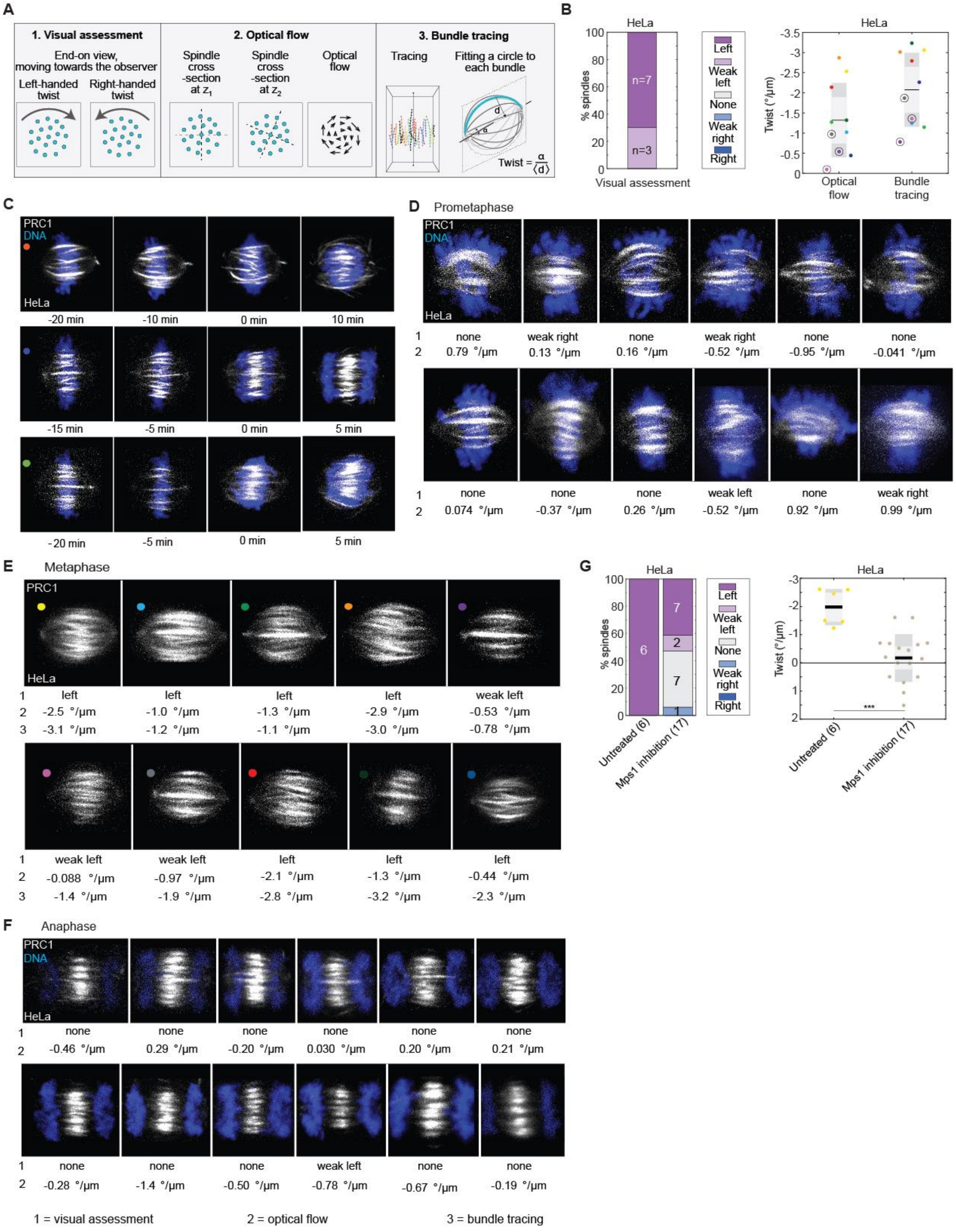

A
1. Visual assessment
End-on view, moving towards the observer
Left-handed twist
Right-handed twist
2. Optical flow
Spindle cross -section at z_1
Spindle cross -section at z_2
Optical flow
3. Bundle tracing
Tracing
Fitting a circle to each bundle
Twist = $\frac{\alpha}{\langle d \rangle}$
B
HeLa
% spindles
n=7
n=3
Visual assessment
Left
Weak left
None
Weak right
Right
Twist (°/μm)
Optical flow
Bundle tracing
C
PRC1
DNA
HeLa
-20 min
-10 min
0 min
10 min
-15 min
-5 min
0 min
5 min
-20 min
-5 min
0 min
5 min
D
Prometaphase
PRC1
DNA
HeLa
1 none / weak right / none / weak right / none / none
2 0.79 °/μm / 0.13 °/μm / 0.16 °/μm / -0.52 °/μm / -0.95 °/μm / -0.041 °/μm
1 none / none / none / weak left / none / weak right
2 0.074 °/μm / -0.37 °/μm / 0.26 °/μm / -0.52 °/μm / 0.92 °/μm / 0.99 °/μm
E
Metaphase
PRC1
HeLa
1 left / left / left / left / weak left
2 -2.5 °/μm / -1.0 °/μm / -1.3 °/μm / -2.9 °/μm / -0.53 °/μm
3 -3.1 °/μm / -1.2 °/μm / -1.1 °/μm / -3.0 °/μm / -0.78 °/μm
1 weak left / weak left / left / left / left
2 -0.088 °/μm / -0.97 °/μm / -2.1 °/μm / -1.3 °/μm / -0.44 °/μm
3 -1.4 °/μm / -1.9 °/μm / -2.8 °/μm / -3.2 °/μm / -2.3 °/μm
F
Anaphase
PRC1
DNA
HeLa
1 none / none / none / none / none / none
2 -0.46 °/μm / 0.29 °/μm / -0.20 °/μm / 0.030 °/μm / 0.20 °/μm / 0.21 °/μm
1 none / none / none / weak left / none / none
2 -0.28 °/μm / -1.4 °/μm / -0.50 °/μm / -0.78 °/μm / -0.67 °/μm / -0.19 °/μm
1 = visual assessment
2 = optical flow
3 = bundle tracing
G
HeLa
% spindles
6
7
2
7
1
Left
Weak left
None
Weak right
Right
Untreated (6)
Mps1 inhibition (17)
Twist (°/μm)
***

Figure S1. Methods for calculating twist and progression of twist during mitosis in HeLa cells. Related to Figure 1.

(A) Schemes of three methods used to measure spindle twist: visual assessment (1), optical flow (2) and bundle tracing (3).

(B) Comparison of the twist for 10 spindles calculated with three different methods. On the left, visual assessment graph represents percentages of spindles showing left, right, weak left, weak right or no twist as described in the legend. On the right, graph shows twist calculated with optical flow and bundle tracing methods; each color represents one cell; circled and un-circled data correspond to the 'weak left' and 'left' data from the visual assessment graph, respectively. Note that the weak left twist in the visual assessment graph corresponds to the range of approximately -1 to -2 °/μm in the bundle tracing method followed along the 5 μm of the bundle length, which corresponds to the rotation of 5-10° in the clockwise direction in the end-on view of the spindle. The black line shows the mean; the light and dark grey areas mark 95% confidence interval on the mean and standard deviation, respectively. Same cells were used to calculate the data for both methods. Experiments were performed on the HeLa-Kyoto BAC cells stably expressing PRC1-GFP (n=10; raw data taken and re-calculated from [S1]).

(C) Microscope images of HeLa cells' spindles in time. Each colored dot represents one spindle's progression through mitosis in time, and each color matches the color of the spindle's data in the graph in Figure 1C. Three individual examples are shown. Microtubule bundles are shown in grey (PRC1-GFP) and DNA in blue (SiR-DNA dye). Images are shown in maximum z-projections. Experiments were performed on the HeLa-Kyoto BAC cells stably expressing PRC1-GFP. Related to Figure 1C.

(D-F) Microscope images of individual **s**pindles of HeLa cells in different phases of mitosis. Examples of spindles in prometaphase (D), metaphase (E) and anaphase (F) are shown with their twist values. Twist was determined with the visual assessment method and the optical flow, marked 1 and 2, respectively. For metaphase spindles twist was also calculated using bundle tracing method, marked 3. Data from these cells was used in the graphs in Figure 1D. Data from metaphase cells was also used in Figure. S1B. Microtubule bundles are shown in grey (PRC1-GFP) and DNA in blue (SiR-DNA dye). Images are shown in maximum z-projections. Experiments were performed on the HeLa-Kyoto BAC cells stably expressing PRC1-GFP. Related to Figure 1B.

(G) Graphs showing twist values after the inhibition of Mps1 in HeLa cells. On the left, visual assessment graph represents percentages of spindles showing left, right, weak left, weak right or no twist as described in the legend. On the right, graph shows twist calculated with the optical flow method. The black line shows the mean; the light and dark grey areas mark 95% confidence interval on the mean and standard deviation, respectively. ***p<0.001 (Student's t-test). Experiments were performed on the HeLa-Kyoto BAC cells stably expressing PRC1-GFP.

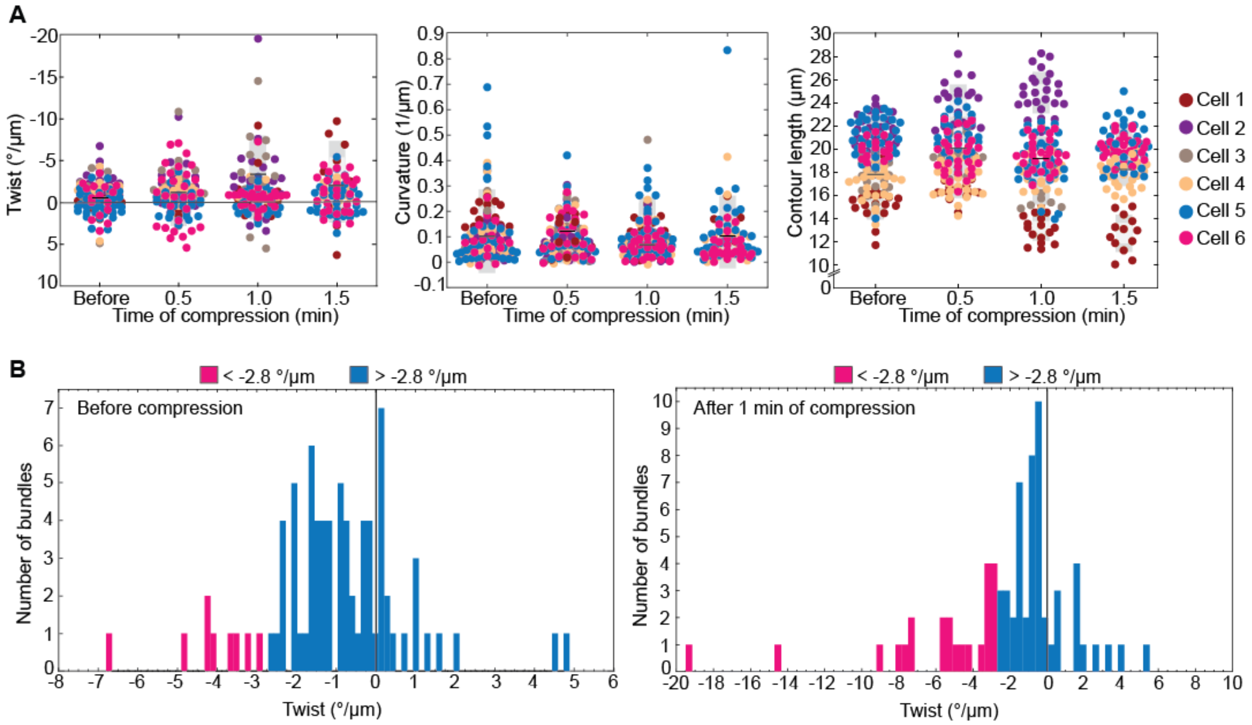

Figure S2. Twist, curvature and contour length of microtubule bundles in spindles compressed by an external force. Related to Figure 2.

(A) On the left, graph show the change of the twist before and up to 1.5 min after the compression. In the middle, graph shows the change of the curvature before and up to 1.5 min after the compression. On the right, graph shows the change of length of the bundle contours before and up to 1.5 min after the compression. Each color represents one cell, as described in the legend; dots represent bundles; the black line shows the mean; the light and dark grey areas mark 95% confidence interval on the mean and standard deviation, respectively. Experiments were performed on the HeLa-Kyoto BAC cells stably expressing PRC1-GFP. Related to Figures 2C-G.

(B) Histograms of twist values before (left) and 1 minute after the compression (right). Colors magenta and blue represent bundles with twist values lower than -2.8 °/µm and above -2.8 °/µm (one standard deviation away from the mean twist before compression), respectively. Note that the distribution shifted towards more negative values upon compression. The twist was smaller than -2.8 °/µm (corresponding to strong left-handed twist) for 9 out of 80 bundles (11.3% ± 3.5%) before compression, whereas after compression this was the case for 21 out of 73 bundles (28.8% ± 5.3%).

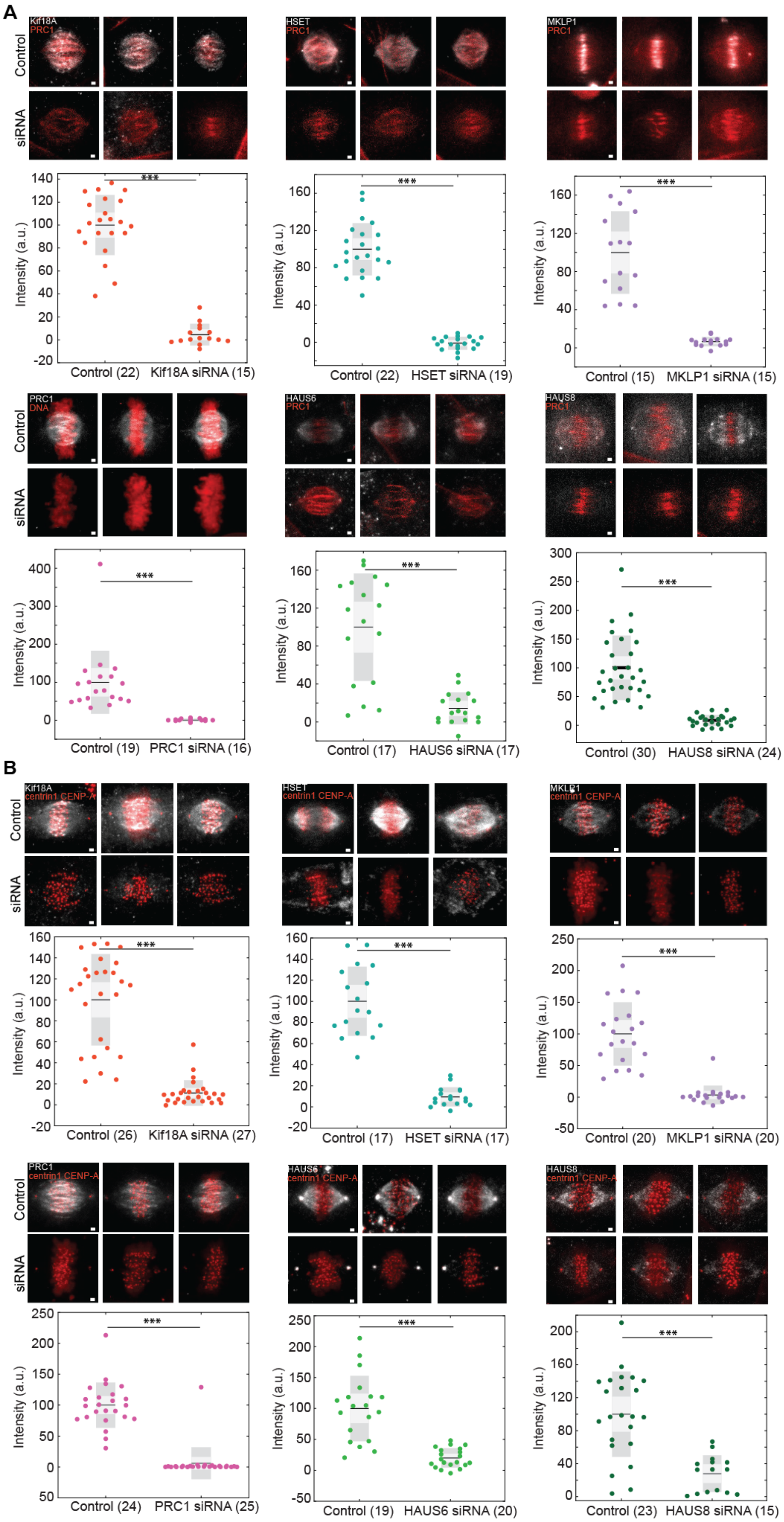
A
Kif18A
PRC1
Control
siRNA
HSET
PRC1
MKLP1
PRC1
Intensity (a.u.)
***
Control (22)
Kif18A siRNA (15)
Control (22)
HSET siRNA (19)
Control (15)
MKLP1 siRNA (15)
PRC1
DNA
HAUS6
PRC1
HAUS8
PRC1
Control (19)
PRC1 siRNA (16)
Control (17)
HAUS6 siRNA (17)
Control (30)
HAUS8 siRNA (24)
B
Kif18A
centrin1 CENP-A
HSET
centrin1 CENP-A
MKLP1
centrin1 CENP-A
Control (26)
Kif18A siRNA (27)
Control (17)
HSET siRNA (17)
Control (20)
MKLP1 siRNA (20)
PRC1
centrin1 CENP-A
HAUS6
centrin1 CENP-A
HAUS8
centrin1 CENP-A
Control (24)
PRC1 siRNA (25)
Control (19)
HAUS6 siRNA (20)
Control (23)
HAUS8 siRNA (15)

Figure S3. Immunofluorescence images of spindles in RPE1 and HeLa cells after protein perturbations. Related to Figures 3 and 4.

(A) Immunofluorescence of HeLa cells' spindles after perturbation of spindle-associated proteins. Examples of three spindles for every perturbation of spindle-associated proteins and their controls: Kif18A depletion, MKLP1 depletion, HSET depletion, PRC1 depletion, HAUS6 and HAUS8 depletion, in that order. Microtubule bundles are shown in grey (proteins of interest) and DNA (dyed with DAPI in non-transfected HeLa) or PRC1 (in HeLa-Kyoto BAC cells stably expressing PRC1-GFP) in red. Images are shown in maximum z-projections. Graphs show intensities of protein of interest in control cells and cells treated with siRNA. ***$p<0.001$ (Student's t-test). Experiments were performed on the non-transfected HeLa cell line (for the depletion of PRC1 and its control) and HeLa-Kyoto BAC cells stably expressing PRC1-GFP (for the rest of the treatments). Related to Figures 3B and 4B.

(B) Immunofluorescence of RPE1 cells' spindles after perturbation of spindle-associated proteins. Examples of three spindles for every perturbation of spindle-associated proteins and their controls: Kif18A depletion, HSET depletion, MKLP1 depletion, PRC1 depletion, HAUS6 and HAUS8 depletion, in that order. Microtubule bundles are shown in grey (proteins of interest) and kinetochores/centrosomes in red. Images are shown in maximum z-projections. Graphs show intensities of protein of interest in control cells and cells treated with siRNA. ***$p<0.001$ (Student's t-test). Experiments were performed on the hTERT-RPE1 cells, permanently transfected and stabilized using CENP-A-GFP and centrin1-GFP. Related to Figures 3C and 4C.

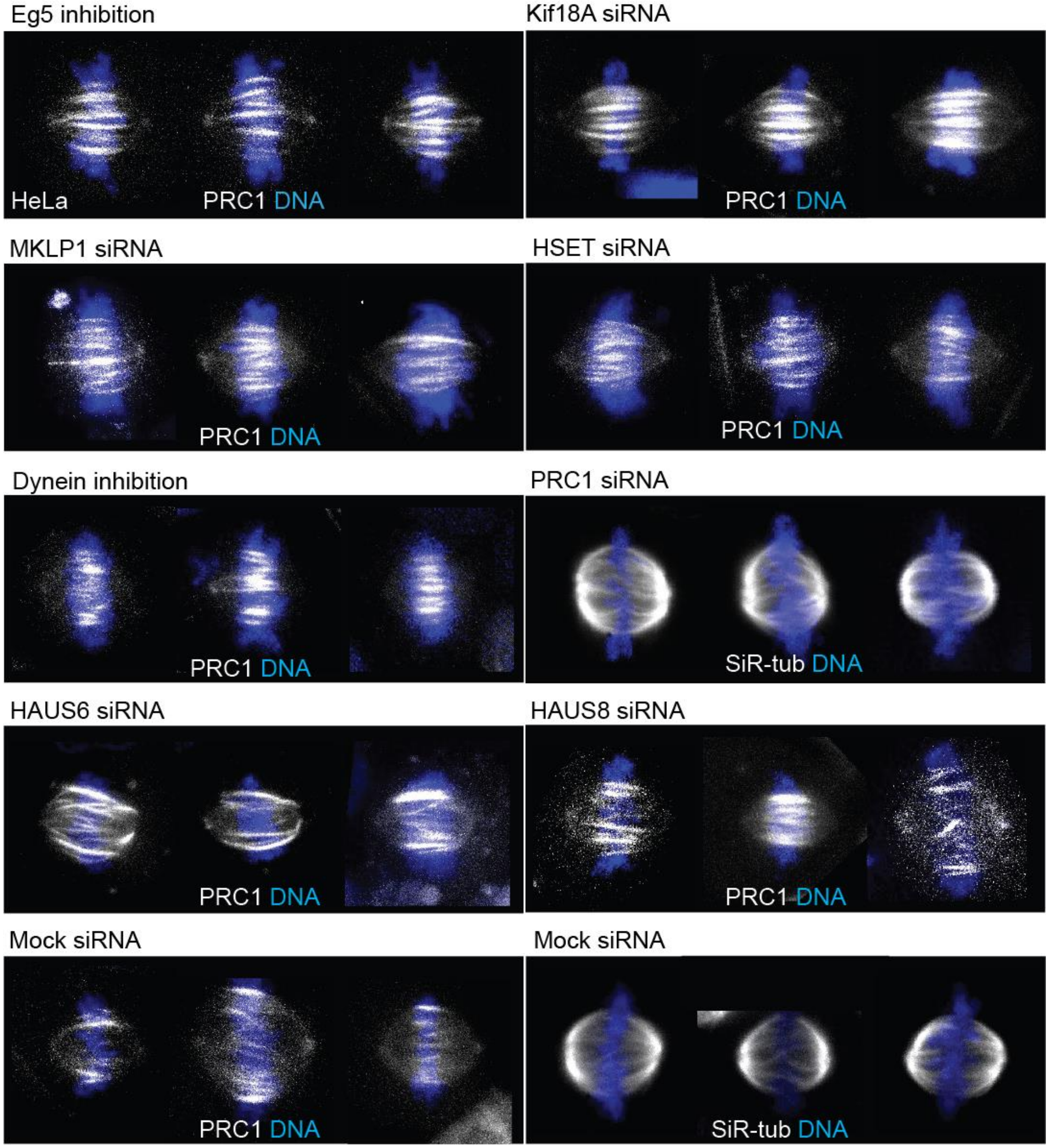
Eg5 inhibition
HeLa
PRC1 DNA
Kif18A siRNA
PRC1 DNA
MKLP1 siRNA
PRC1 DNA
HSET siRNA
PRC1 DNA
Dynein inhibition
PRC1 DNA
PRC1 siRNA
SiR-tub DNA
HAUS6 siRNA
PRC1 DNA
HAUS8 siRNA
PRC1 DNA
Mock siRNA
PRC1 DNA
Mock siRNA
SiR-tub DNA

Figure S4. Spindles of HeLa cells after perturbation of spindle-associated proteins. Related to Figures 3 and 4.

Examples of three spindles for every perturbation of spindle-associated proteins: Eg5 inhibition, Kif18A depletion, MKLP1 depletion, HSET depletion, dynein inhibition, PRC1 depletion, HAUS6 and HAUS8 depletion and mock controls. Data was used in the graphs in Figures. 3B and 4B. Microtubule bundles are shown in grey (SiR-tubulin in non-transfected HeLa and PRC1-GFP in HeLa-Kyoto BAC cell line) and DNA in blue (SiR-DNA dye in HeLa-Kyoto BAC cell line and NucBlue dye in non-transfected HeLa). Images are shown in maximum z-projections. Experiments were performed on the non-tranfected HeLa cell line and HeLa-Kyoto BAC cells stably expressing PRC1-GFP. Related to Figures 3A and 4A.

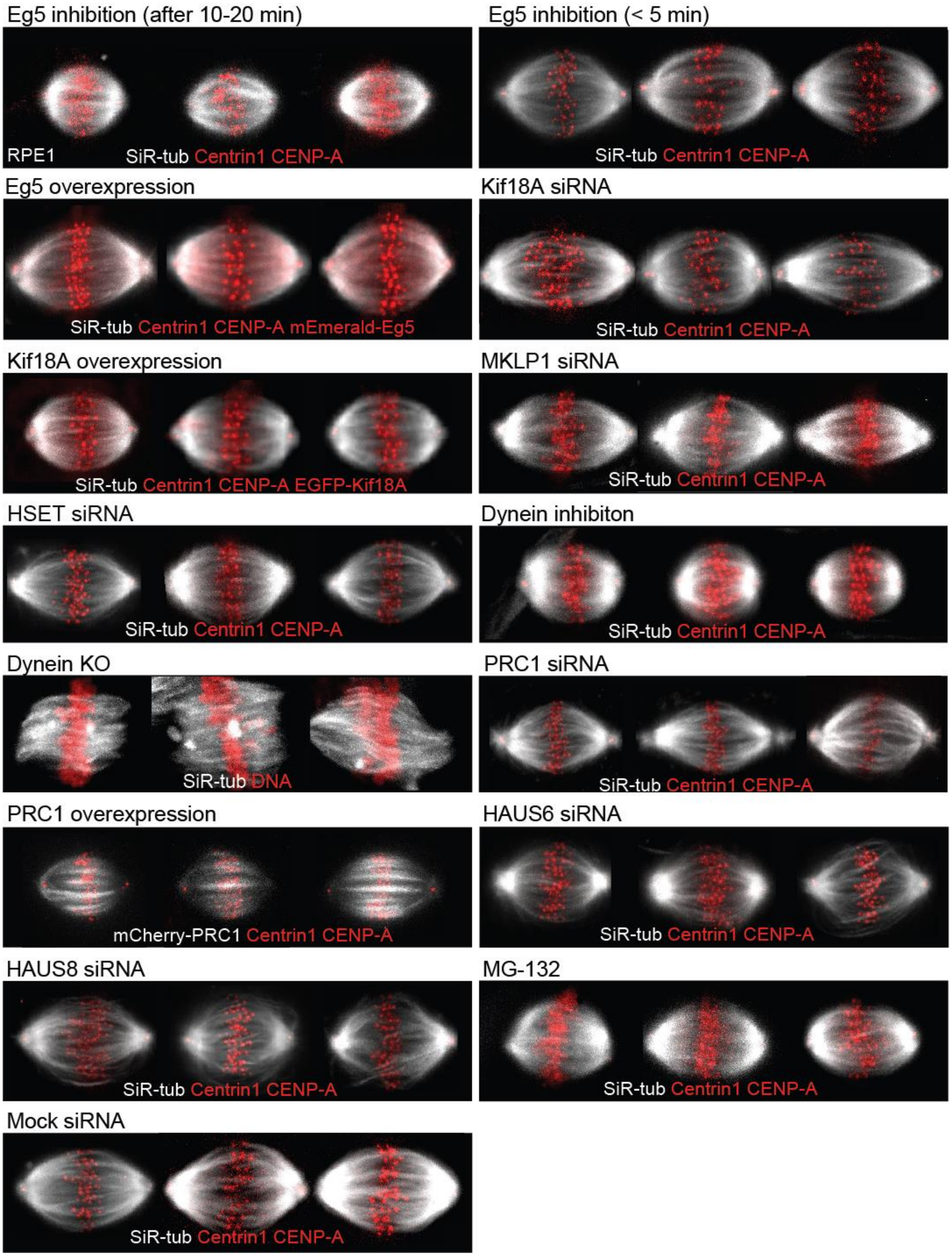
Eg5 inhibition (after 10-20 min)
RPE1
SiR-tub Centrin1 CENP-A
Eg5 inhibition (< 5 min)
SiR-tub Centrin1 CENP-A
Eg5 overexpression
SiR-tub Centrin1 CENP-A mEmerald-Eg5
Kif18A siRNA
SiR-tub Centrin1 CENP-A
Kif18A overexpression
SiR-tub Centrin1 CENP-A EGFP-Kif18A
MKLP1 siRNA
SiR-tub Centrin1 CENP-A
HSET siRNA
SiR-tub Centrin1 CENP-A
Dynein inhibiton
SiR-tub Centrin1 CENP-A
Dynein KO
SiR-tub DNA
PRC1 siRNA
SiR-tub Centrin1 CENP-A
PRC1 overexpression
mCherry-PRC1 Centrin1 CENP-A
HAUS6 siRNA
SiR-tub Centrin1 CENP-A
HAUS8 siRNA
SiR-tub Centrin1 CENP-A
MG-132
SiR-tub Centrin1 CENP-A
Mock siRNA
SiR-tub Centrin1 CENP-A

Figure S5. Spindles of RPE1 cells after perturbation of spindle-associated proteins. Related to Figures 3 and 4.

Examples of three spindles for every perturbation of spindle-associated proteins: Eg5 inhibition (after 10-20 min of STLC treatment), Eg5 inhibition (STLC treatment shorter than 5 min), Eg5 overexpression, Kif18A depletion, Kif18A overexpression, MKLP1 depletion, HSET depletion, dynein inhibition, dynein KO, PRC1 depletion, PRC1 overexpression, HAUS6 and HAUS8 depletion, MG-132 treatment, and mock control. Data was used in the graphs in Figures 3C and 4C. Microtubule bundles are shown in grey (SiR-tubulin and, for PRC1 overexpression, mCherry-PRC1) and kinetochores/centrosomes, DNA (NucBlue dye), Eg5 and KiF18A in red. Images are shown in maximum z-projections. Experiments were performed on hTERT-RPE1 cells, permanently transfected and stabilized using CENP-A-GFP and centrin1-GFP and RPE1 inducible CRISPR/Cas9 DYNC1H1 knockout cells. Related to Figures 3A and 4A.

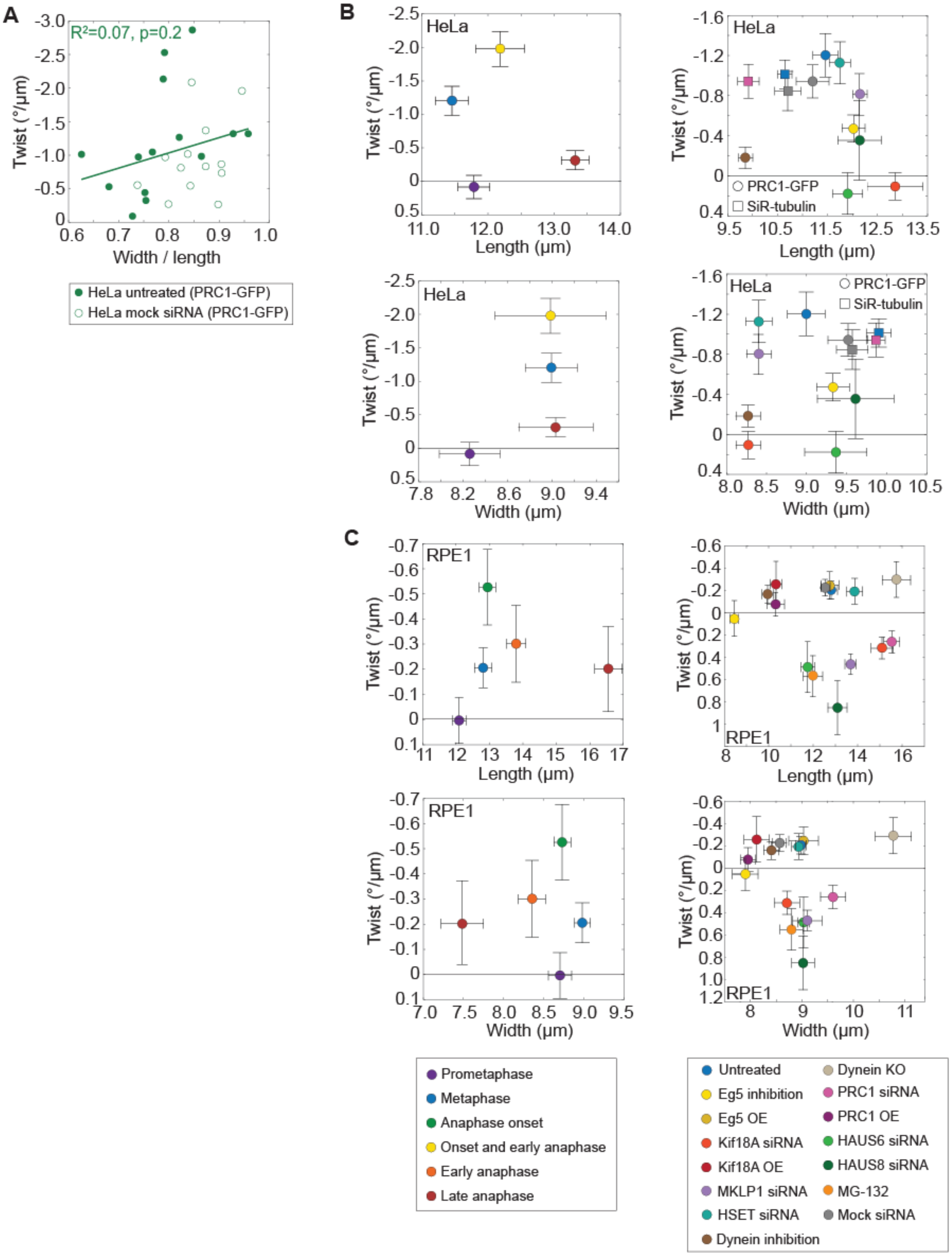
A
R²=0.07, p=0.2
Twist (°/µm)
Width / length
HeLa untreated (PRC1-GFP)
HeLa mock siRNA (PRC1-GFP)
B
HeLa
Twist (°/µm)
Length (µm)
Width (µm)
PRC1-GFP
SiR-tubulin
C
RPE1
Twist (°/µm)
Length (µm)
Width (µm)
Prometaphase
Metaphase
Anaphase onset
Onset and early anaphase
Early anaphase
Late anaphase
Untreated
Eg5 inhibition
Eg5 OE
Kif18A siRNA
Kif18A OE
MKLP1 siRNA
HSET siRNA
Dynein inhibition
Dynein KO
PRC1 siRNA
PRC1 OE
HAUS6 siRNA
HAUS8 siRNA
MG-132
Mock siRNA

Figure S6. Correlation between spindle length or width and spindle twist in different phases of mitosis and during different protein perturbations in HeLa and RPE1 cells. Related to Figure 5.

(A) Round spindles have stronger twist than elongated spindles. Graph shows the correlation between width/length ratio and twist in HeLa cells. Filled circles represent untreated cells while empty circles represent mock siRNA controls. Lines show linear fit (untreated cells together with mock siRNA controls); equation y=-2.28x+0.79; goodness of fit shown in the graph. Same data was used in Figures. 1D, 3B and 4B. Experiments were performed on the HeLa-Kyoto BAC cells stably expressing PRC1-GFP. Related to Figure 5A.

(B) Graphs showing how the spindle twist and length (top) or width (bottom) in HeLa cells vary depending on the different phases of mitosis (left) or perturbations of spindle-associated proteins (right). Each color represents different phase of mitosis or protein perturbation, as described in the legend at the bottom; error bars represent SEM. Same data was used in graphs in Figures. 1D, 3B and 4B. Experiments were performed on the HeLa-Kyoto BAC cells stably expressing PRC1-GFP (dots) and non-tranfected HeLa cell line for the depletion of PRC1 and its controls (rectangles). Related to Figures 5B and 5C.

(C) Graphs showing how the spindle twist and length (top) or width (bottom) in RPE1 cells vary depending on the different phases of mitosis (left) or perturbations of spindle-associated proteins (right). Each color represents different phase of mitosis or protein perturbation, as described in the legend at the bottom; error bars represent SEM. Same data was used in graphs in Figures 1E, 3C and 4C. Experiments were performed on hTERT-RPE1 cells, permanently transfected and stabilized using CENP-A-GFP and centrin1-GFP and RPE1 inducible CRISPR/Cas9 DYNC1H1 knockout cells. Related to Figures 5B and 5C.

SUPPLEMENTAL REFERENCE

S1. Novak, M., Polak, B., Simunic, J., Boban, Z., Kuzmic, B., Thomae, A.W., Tolic, I.M., and Pavin, N. (2018). The mitotic spindle is chiral due to torques within microtubule bundles. Nat Commun 9, 3571.
